# Targeting USP18 overcomes acquired resistance in hepatocellular carcinoma by regulating NCOA4 deISGylation and ferroptosis

**DOI:** 10.1038/s41419-025-07772-0

**Published:** 2025-06-13

**Authors:** Shengtao Ye, Junxin Chen, Ying Zheng, Mengmeng He, Yanqiu Zhang, Yang Cheng, Yingrong Leng, Enyi Wu, Lingyi Kong, Hao Zhang

**Affiliations:** Jiangsu Key Laboratory of Bioactive Natural Product Research and State Key Laboratory of Natural Medicines, School of Traditional Chinese Pharmacy, China Pharmaceutical University, Nanjing, China

**Keywords:** Cancer therapeutic resistance, Post-translational modifications

## Abstract

Targeted therapy resistance has become a major challenge for hepatocellular carcinoma (HCC) treatment. Triggering ferroptosis emerges as a promising strategy to overcome therapeutic resistance. Here, we have identified ubiquitin-specific protease 18 (USP18), a member of the deubiquitinating enzyme family, contributing to HCC resistance by inhibiting sorafenib-induced ferroptosis. Nuclear receptor coactivator 4 (NCOA4), a crucial regulator of ferroptosis, turned out to be a novel downstream effector of USP18 and is posttranslationally suppressed. Such regulation is based on the USP18-mediated deISGylation and degradation process. Additionally, we have demonstrated that sorafenib promotes USP18 accumulation in HCC via the STING/IRF3/ISG15 axis. Importantly, we screened and identified hyperoside (HYP) as a new USP18 enzyme activity inhibitor, which sensitizes cancer cells to existing targeted therapies (sorafenib and regorafenib) by inhibiting USP18 and following deISGylation of NCOA4. Collectively, our study has uncovered a novel mechanism of acquired sorafenib resistance and offers a promising combination therapy strategy for overcoming therapeutic resistance in HCC.

## Introduction

Hepatocellular carcinoma (HCC) is the fifth most common malignant tumor in the world and the third leading cause of cancer-related death, constituting 70%–90% of primary liver cancer cases [1]. Sorafenib is the first multi-targeted tyrosine kinase inhibitor approved by the FDA for the treatment of advanced-stage liver cancer. Unfortunately, the clinical effectiveness of sorafenib on HCC has been greatly limited after 4–5 months of treatment due to the occurrence of primary or acquired drug resistance [2]. Although there is increasing awareness of sorafenib resistance in HCC, comprehending its underlying molecular mechanisms remains intricate and largely enigmatic [3]. There is an urgent need for further investigation into the molecular mechanisms of sorafenib resistance, which could aid in identifying new targets for rational combination therapy to overcome this formidable obstacle [4].

Ubiquitin-specific protease 18 (USP18), also known as UBP43, is a member of the deubiquitinating (DUB) enzyme family and plays a pivotal role in various pathological conditions, including viral and bacterial infections, autoimmune diseases, neurological disorders, and tumor progression [5–7]. Previous studies have primarily focused on elucidating the regulatory function of USP18 in antiviral and antibacterial infection responses by inhibiting the type I interferon (IFN) signaling pathway independently of its catalytic activity [8, 9]. In recent years, compelling evidence suggests that USP18 functions as a deconjugating enzyme responsible for the removal of ISG15 from substrate proteins (deISGylation), thereby playing an indispensable role in tumorigenesis and development [10]. USP18 expression is upregulated in HCC patients [11], however, the role of USP18 in acquired sorafenib resistance of HCC remains unexplored.

ISG15, the first identified member of the ubiquitin-like protein family, has been extensively studied for its role in ISGylation. Notably, various molecules including cyclic GMP-AMP synthase (cGAS) [12], tumor susceptibility gene 101 (TSG101) [13], NOD-like receptor thermal protein domain associated protein 3 (NLRP3) [14], and signal transducer and activator of transcription 1 (STAT1) [15] have been confirmed to undergo ISGylation. Unlike ubiquitylation mediated by the Lys48 bond, ISGylation can competitively bind to ubiquitin-binding sites on a protein and subsequently inhibit the degradation of ubiquitin substrates [14, 16, 17]. ISGylation has been proven to be widely involved in human immunity, viral infection, and tumor development [18, 19]. Nevertheless, the potential effects of ISGylation on targeted therapy resistance of HCC have not been investigated yet.

Ferroptosis represents a distinct form of programmed cell death, characterized by unique genetic processes, biochemical activities, and morphological characteristics that set it apart from necrosis, apoptosis, and autophagy [20]. Ferroptosis inhibitors, such as Ferrostatin-1, Rosiglitazone (an ACSL4 inhibitor), or Desferoxamine (an iron ion chelator), possess the ability to impede the anticancer activity of sorafenib [21, 22]. Furthermore, increasing evidence highlights the potential therapeutic value of targeting ferroptosis to overcome sorafenib resistance in HCC [23–25]. Iron governs ferroptosis not only by initiating the nonenzymatic Fenton reaction for direct peroxidation of PUFA-PLs but also by serving as an indispensable cofactor for enzymes involved in lipid peroxidation (such as ALOX and POR) [26, 27]. Nuclear receptor coactivator 4 (NCOA4) functions as a selective cargo receptor for ferritinophagy, playing a crucial role in maintaining iron homeostasis. Inhibition of NCOA4-mediated ferritinophagy leads to a reduction in the labile iron pool and suppression of ferroptosis [28]. However, the precise mechanisms underlying the involvement of NCOA4 in both the effectiveness and resistance to targeted therapy remain largely unknown and require further elucidation.

In this study, we have discovered a previously unrecognized function of USP18 in acquired resistance to sorafenib in HCC. Mechanistic investigations have elucidated that sorafenib-induced STING/IRF3/ISG15 axis activation leads to USP18 accumulation and resistance. USP18 diminished the stability of NCOA4 protein by promoting NCOA4 deISGylation and degradation and impedes NCOA4-mediated ferroptosis. We screened and identified hyperoside (HYP) as a new USP18 inhibitor, which exhibits remarkable potential in augmenting the therapeutic efficacy of sorafenib and regorafenib in liver tumors. Our study presents a promising therapeutic approach to overcome targeted therapy resistance in HCC by targeting USP18.

## Materials and methods

### Human HCC samples

The human HCC tissue microarrays (LVC1609), consisting of 80 pairs of HCC tissues with comprehensive clinicopathological and follow-up data, were purchased from Shanghai LIAODING Biotech (Shanghai, China). The vendor provided clinical information, including sex, age, pathological diagnosis, disease-free survival, and overall survival. The inclusion criteria and clinical status of all patients were:

- Definitive HCC diagnosis by pathology.
- Surgical resection is defined as complete resection of all tumor nodules, with the cut surface being free of cancer by histological examination.
- The patients received treatment with sorafenib after surgery.

### Animal experiments

All animal care and experimental procedures were approved by the University Committee on Use and Care of Animals at China Pharmaceutical University (Nanjing, China) under Resolution Number 2022-03-022. The animal studies conducted in this research adhere to the ARRIVE guidelines. Four-week-old male C57BL/6J mice (12–17 g, specific pathogen-free class) and four-week-old male Balb/c nude mice (12–16 g, specific pathogen-free class) were procured from Beijing Vital River Laboratory Animal Technology Co., Ltd (Beijing, China). All animals were housed in a controlled environment room maintained at a temperature range of 20–24 °C with relative humidity between 40% and 60%, following a 12 h light/dark cycle. Random allocation was employed for grouping the mice into equal-sized cohorts, while analysis of all animal samples was performed in a blinded manner.

To establish an HCC xenograft model, subcutaneous injections of 6 × 10^6^ HepG2-Ctrl or HepG2-USP18-OE cells/200 μL serum-free DMEM and Matrigel (1:1) were administered to each nude mouse. After approximately four weeks, when the tumor volume reached around 100 mm^3^, mice were subjected to the indicated treatment. The size of the subcutaneous tumors was measured and recorded every 3 d using Vernier calipers according to the formula: tumor volume (mm^3^) = (L × W^2^)/2, where L represents the long axis and W denotes the short axis. After completing the treatment, all mice were euthanized, and their tumors were excised for subsequent analysis.

For the hydrodynamic injection model, a mixture of N-Ras plasmid (1.90 μg/g), c-Myc plasmid (0.10 μg/g), and sleeping beauty transposase (SBT) (0.2 μg/g) was diluted in saline solution (0.9% NaCl), filtered through a 0.22 μm filter, and injected into the lateral tail vein of C57BL/6J mice within 5–7 s. Following administration of the proto-oncogene via tail vein injection for 3.5 w, treatment with sorafenib at a dosage of 30 mg/kg/d was initiated for an additional 2.5 w to mimic the clinical scenario where patients with HCC who are nonresponsive to sorafenib experience disease progression after its administration. The mice were subjected to the indicated treatment. After completing the treatment, all mice were sacrificed, and their livers were collected for further investigations. A portion of the liver tissue samples was fixed with 4% paraformaldehyde for subsequent histological examination, while the remaining samples were stored at −80 °C until further analysis.

### Label-free proteomics analysis

During the protein extraction process, frozen samples underwent ultrasonic disruption (80 W power, ultrasound on for 1.0 s/off for 1.0 s, lasting for 3 min) followed by treatment with lysis buffer containing 1 mM PMSF. Total proteins were obtained through centrifugation at 12,000 × *g* (at room temperature for 10 min). After quantitation using the BCA method, the proteins were aliquoted and stored frozen at −80 °C. For trypsin digestion, 50 μg of protein was first reduced with 5 mM DTT (at 55 °C for 30 min) and then alkylated with 10 mM iodoaceta-mide (at room temperature for 15 min). This was followed by acetone precipitation (−20 °C overnight) and resolubilization in 200 mM TEAB. 1 mg/ml of trypsin (1:50 w/w) was added for digestion at 37 °C overnight, and the resulting peptides were lyophilized and stored. For peptide labeling, TMT reagents were used: the lyophilized peptides were resolubilized in 100 mM TEAB and reacted with activated TMT reagents (dissolved in 88 μL of acetonitrile) at room temperature for 1 h. The reaction was terminated with hydroxylamine and then lyophilized. Reverse phase chromatographic separation was performed using an Agilent 1100 HPLC system with a Zorbax Extend-C18 column (2.1 × 150 mm, 5 μm). Gradient elution was carried out with a pH 10 ACN-H_2_O buffer (0–75 min), and fractions from 8–60 min were collected and lyophilized. Mass spectrometry analysis was conducted using a Thermo Fisher Q Exactive HF-X, employing nano-flow LC–MS/MS via a PepMap RSLC column (75 μm × 50 cm). Gradient elution was performed (0–60 min, 8–85% B phase) with a primary resolution of 70,000. The top 10 parent ions were dynamically selected for HCD fragmentation (energy 32), and the secondary resolution was set at 17,500. Data were analyzed using Proteome Discoverer 2.4, and differential proteins were identified in combination with the UniProt database.

The peptides underwent LC–MS analysis, and label-free quantification was employed to compare protein abundance between the HepG2-USP18-OE and HepG2-Ctrl groups. To identify significant differences, a Student’s t-test was applied. Peptides meeting the following criteria were considered differentially expressed: (1) fold changes > 1.2 and (2) p_value_ < 0.05. The mass spectrometry proteomics data have been deposited in the ProteomeXchange Consortium (http://proteomecentral.proteomexchange.org) via the iProX partner repository with the dataset identifier PXD047888.

### Biolayer interferometry (BLI) assay

The dose-dependent binding affinities of HYP for USP18 WT and USP18 IBB1 MUT were determined using a BLI assay conducted on Octet RED96 (ForteBio). Ni-NTA biosensor tips (ForteBio, Menlo Park, CA) were utilized to immobilize the His-labeled proteins after pre-wetting with kinetic buffer (PBST, 0.05% bovine serum albumin, 0.01% Tween 20). The equilibrated Ni-NTA biosensors were loaded with either USP18 WT or USP18 IBB1 MUT. Duplicate sets of sensors incubated in a buffer without proteins served as background binding controls. All assays adhered to a standard protocol and were performed in 96-well black plates at a constant volume of 250 μL/well, maintained at 30°C. Data analysis was carried out using Octet data analysis software, employing a double reference subtraction protocol to account for nonspecific and background signals as well as signal drifts caused by biosensor variability. Equilibrium dissociation constant (K_D_) values were calculated based on the ratio of K_off_ to K_on_.

### Data, materials, and software availability

The results are presented as means ± SEM. In vivo experiments were designed to establish equal size, blinding, and randomization. The statistical analyses were performed only for experiments where group sizes (*n*) ≥5. All group sizes represent the number of experimental independent values, and these independent values were used to evaluate statistical analyses. The in vitro experiment was set up to use at least 3 samples (biological replicates) per experiment/group/condition. For survival analysis, the expression of indicated targets was treated as a binary variant and divided into ‘high’ and ‘low’ levels. Kaplan–Meier survival curves were compared using the Log-rank (Mantel-Cox) test. All statistical analysis was performed using GraphPad Prism 8.0 (GraphPad Software, Inc., La Jolla, CA, USA; RRID: SCR_002798). Statistical differences between the two groups were analyzed by the unpaired Student’s t-test with a two-tailed distribution. Differences between multiple groups of data were analyzed by one-way ANOVA with Bonferroni correction. p_value_ less than 0.05 were considered statistically significant.

Part of the materials and methods are described in the supplementary file.

## Results

### DUB siRNA library screening identifies USP18 as a key driver of sorafenib resistance

**Fig. 1:**
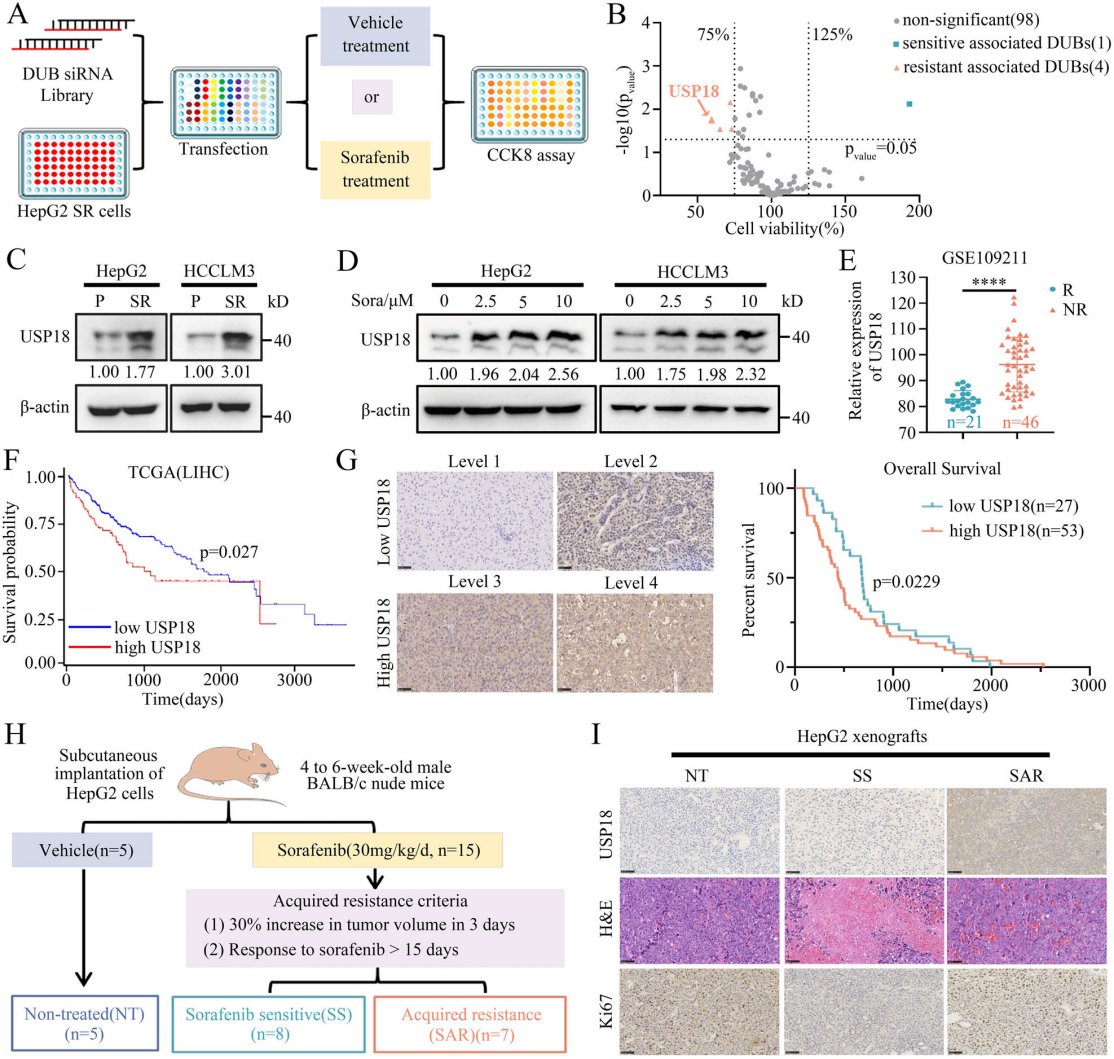
DUB siRNA library screening identifies USP18 as a key driver of sorafenib resistance. **A** The schematic diagram illustrates the workflow of the DUB siRNA library screening strategy used in this study. **B** Volcano map characterization screening results. The rose red triangles indicate resistance-associated DUBs (p < 0.05 and cell viability <75%). Blue-gray squares indicate sensitive associated DUBs (p < 0.05 and cell viability >125%). The cell viability was determined by comparing it to the control group, which consisted of cells treated with sorafenib alone. **C** Protein expression of USP18 in parental (P) and sorafenib-resistant (SR) cells. The intensities of bands were analyzed by Image J and normalized to the corresponding parental (P) cells. **D** Representative immunoblot images of USP18 in HepG2 and HCCLM3 cells treated with sorafenib (0, 2.5, 5, and 10 μM) for 24 h. The band intensities were quantified using Image J and normalized to the control cells treated with DMSO. **E** The USP18 levels in HCC tissue samples of non-responders (NR) and responders (R) to sorafenib treatment were assessed using the GSE109211 dataset (means ± SEM, ****p < 0.0001, unpaired Student’s t-tests). **F** The Kaplan–Meier curves demonstrate the association between USP18 expression and overall survival among HCC patients in the TCGA cohort. **G** Representative IHC images of USP18 at different staining intensity levels (levels 1–4 represent progressive low to high staining) in resected HCC samples from patients who went on to receive sorafenib treatment (left). Kaplan–Meier survival analysis comparing the cumulative survival rate of patients with different USP18 expression levels (n = 80 cases) (right). Patients were distinguished by the median expression level of USP18, using the Log-rank (Mantel-Cox) test. Scale bars, 100 μm. **H** Schematic overview of an HCC xenograft model that acquired resistance to sorafenib. **I** Representative H&E staining images and IHC images of USP18 and Ki67 in excised xenografts. Scale bars, 100 μm.

To mimic the biological process of sorafenib resistance in HCC patients, we established two HCC sorafenib-resistant cell lines, named HepG2-SR and HCCLM3-SR, by culturing cells with escalating doses of sorafenib over 24 weeks (Supplementary Fig. S1A). We then confirmed the sorafenib resistance of the two cell lines employing CCK-8, EDU, and colony formation assay (Supplementary Fig. S1B–D). To investigate potential DUB enzyme involved in sorafenib resistance, we conducted a comprehensive DUB enzyme siRNA library screening (Fig. 1A). Among them, USP18 knockdown significantly enhanced the susceptibility of HCC-SR cells (Fig. 1B). USP18 expression increased in HCC-SR cells (Fig. 1C), as well as in the parental HepG2 and HCCLM3 cells following sorafenib treatment (Fig. 1D). In addition, the analysis of the GSE109211 dataset revealed a consistent upregulation of USP18 in tumor tissue samples obtained from HCC patients who exhibited a non-responsive phenotype towards sorafenib treatment (Fig. 1E). Moreover, Kaplan–Meier analysis of the TCGA dataset demonstrated a significant correlation between elevated USP18 expression levels and unfavorable overall survival outcomes among HCC patients (Fig. 1F). To further validate our findings, we analyzed by USP18 immunohistochemistry 80 HCCs from patients who underwent sorafenib treatment following surgery. Reduced immunostaining of USP18 was associated with improved patient survival following treatment with sorafenib (*p* < 0.05, 439 vs. 686 days) (Fig. 1G). The findings above suggest that USP18 may play a crucial role in developing sorafenib resistance and serve as a valuable biomarker for the response of patients to sorafenib.

To further validate the results, we generated acquired resistant models through in vivo xenograft tumor establishment followed by sorafenib treatment (Fig. 1H). The mice were categorized into non-treated (NT), sorafenib-sensitive (SS), and acquired sorafenib-resistant (SAR) groups, employing established criteria for tumor volume changes (Supplementary Fig. S1E, F) [29]. The SR group exhibited accelerated tumor growth and cell proliferation as indicated by tumor weight, H&E, and Ki67 staining (Fig. 1I and Supplementary Fig. S1G). As anticipated, the positive correlation between USP18 expression and acquired drug resistance was further validated by IHC and western blot analysis (Fig. 1I and Supplementary Fig. S1H). Taken together, these findings indicate that the accumulation of USP18 may play a critical role in the emergence of sorafenib resistance in HCC.

### USP18 promotes HCC resistance to sorafenib in vitro and in vivo

**Fig. 2:**
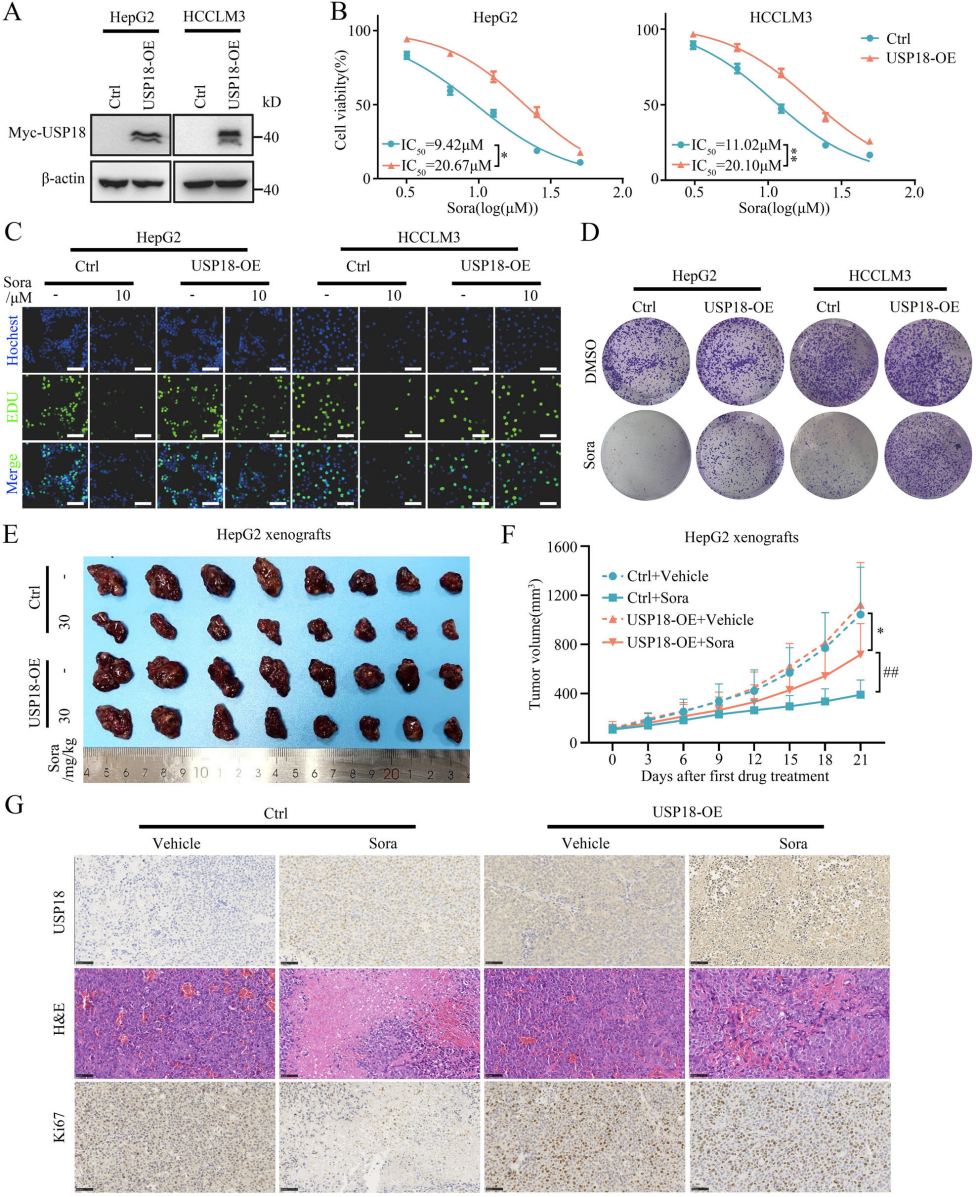
USP18 promotes HCC resistance to sorafenib in vitro and in vivo. **A** The efficacy of USP18 overexpression was validated in two USP18 overexpression stable cell lines (HepG2-USP18-OE and HCCLM3-USP18-OE). **B**–**D** CCK-8 (**B**), EDU (**C**), and clone formation assay (**D**). The impact of USP18 overexpression on the susceptibility of HCC cells toward sorafenib treatment (means ± SEM, *p < 0.05, **p < 0.01, paired student’s t-test). Scale bars, 5 μm. **E** Representative pictures of subcutaneous HepG2 xenografts from the indicated groups. n = 8 mice per group. **F** The tumor growth curve of HepG2-USP18-OE cells or HepG2-Ctrl cells in nude mice and treated with vehicle or sorafenib. Tumor volume was measured every 3 days beginning from the first treatment (means ± SEM, *p < 0.05 versus Ctrl + Vehicle, ^##^p < 0.01 versus Ctrl + Sora, one-way ANOVA test). n = 8 mice per group. **G** Representative H&E staining images and IHC images of USP18 and Ki67 in excised xenografts. Scale bars, 100 μm.

To study whether USP18 confers resistance of HCC cells to sorafenib, we generated two stable USP18 overexpression cell lines (HepG2-USP18-OE and HCCLM3-USP18-OE) (Fig. 2A). We found that USP18 overexpression significantly attenuated the susceptibility of HCC cells to sorafenib treatment as evidenced by CCK-8, EDU, and clone formation assay (Fig. 2B–D). Conversely, USP18 knockdown enhanced the cell-killing effect of sorafenib on HCC-SR cells (Supplementary Fig. S2A–D). Notably, neither USP18 overexpression nor knockdown alone exerted any significant effect on HCC cell proliferation.

To further validate the roles of USP18 in vivo, HepG2 cells with or without USP18 overexpression were injected into nude mice. Once tumors became detectable (tumor volume ≥100 mm^3^), mice were treated with sorafenib by oral gavage. Consistent with the in vitro findings, USP18 overexpression showed no significant effect on tumor growth; however, it decreased the inhibitory effect of sorafenib on tumor growth (Fig. 2E). The tumors from USP18 overexpression mice were significantly larger than those from control mice upon treatment with sorafenib, indicating an enhanced drug resistance in the USP18 overexpression group (Fig. 2F and Supplementary Fig. S2E). Similarly, IHC staining results showed that USP18 overexpression compromised the efficacy of sorafenib in inhibiting tumor cell proliferation (Fig. 2G). Taken together, these findings indicate that USP18 confers HCC cell resistance to sorafenib in vitro and in vivo.

### USP18 promotes resistance by antagonizing sorafenib-induced ferroptosis

**Fig. 3:**
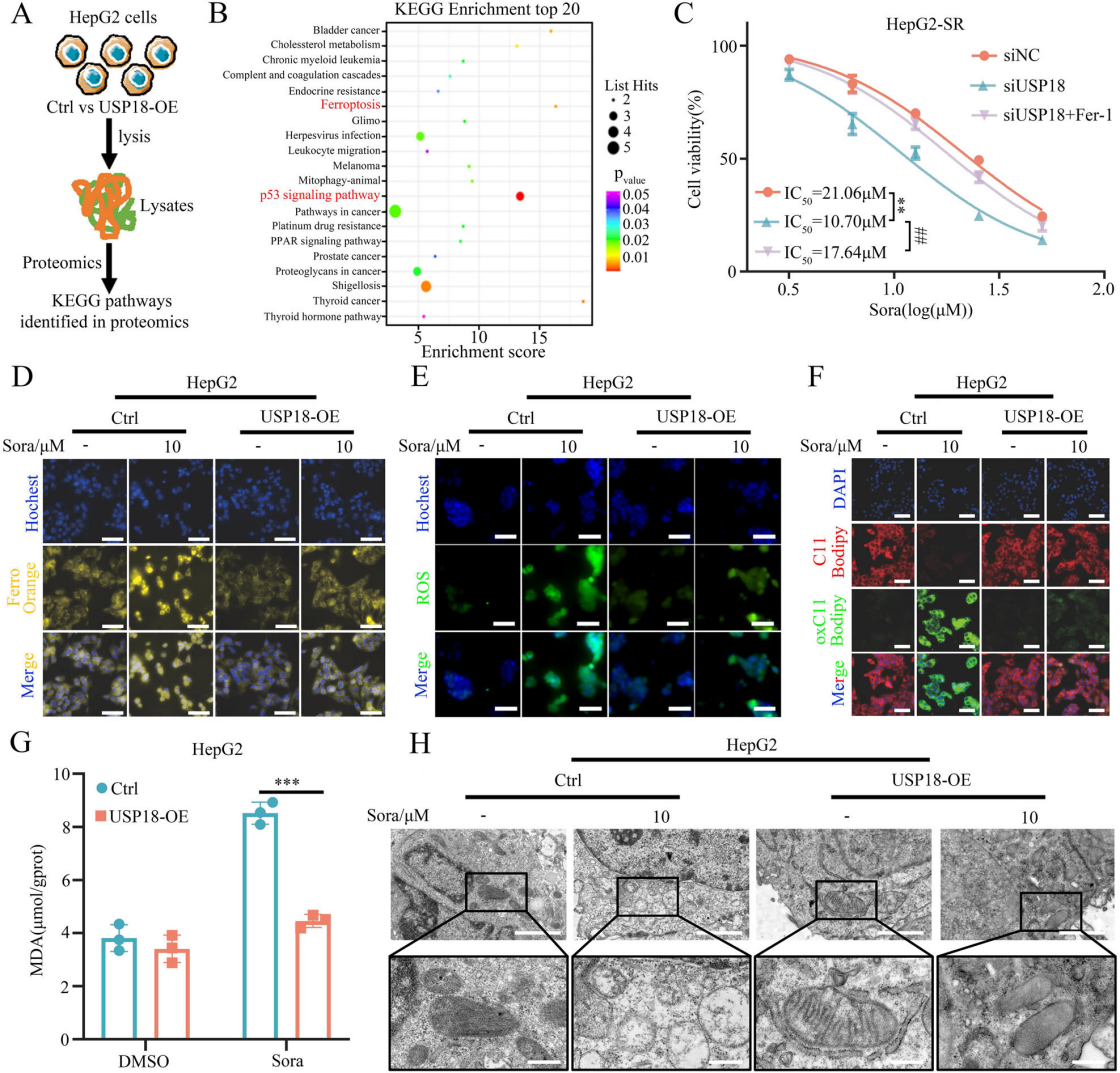
USP18 promotes resistance by antagonizing sorafenib-induced ferroptosis. **A** Scheme displaying the procedure used for identifying biological processes regulated by USP18. **B** The bubble diagram of KEGG pathway analysis of the top 20 biological processes downregulated by USP18. **C** CCK-8 assay. USP18 knockdown on the susceptibility to sorafenib in HepG2-SR cells, in the presence or absence of ferroptosis inhibitor (Fer-1, 2 μM) treatment for 24 h (means ± SEM, **p < 0.01 versus siNC, ^##^p < 0.01 versus siUSP18, paired student’s t-test). **D** FerroOrange staining. The impact of USP18 overexpression on the sorafenib-induced elevation of Fe^2+^ levels. Scale bars, 5 μm. **E** ROS staining. The effect of USP18 overexpression on the generation of ROS induced by sorafenib. Scale bars, 5 μm. **F**, **G** BODIPY 581/591 C11 staining (**F**) and MDA assay (**G**). The impact of USP18 overexpression on sorafenib-induced lipid peroxidation (means ± SEM, ***p < 0.001, student’s t-test). Scale bars, 5 μm. **H** Representative transmission electron microscopy (TEM) images of mitochondrial morphological changes in the indicated group. Scale bars, 2 µm (top), 500 nm (bottom).

To elucidate the molecular mechanism underlying USP18-mediated acquired resistance, we conducted a comprehensive proteomic analysis comparing HepG2-USP18-OE cells with HepG2-Ctrl cells (Fig. 3A). Subsequent KEGG pathway enrichment analysis indicated that ferroptosis and p53 signaling pathways were significantly altered under USP18 overexpression (Fig. 3B). Notably, the p53 signaling pathway also involved in ferroptosis [30]. This prompted us to investigate whether USP18-mediated sorafenib resistance through regulating ferroptosis. Indeed, the inhibitory effect of sorafenib on HCC cell proliferation was markedly inhibited by the ferroptosis inhibitor, Ferrostatin-1 (Fer-1) (Supplementary Fig. S3A). Moreover, the USP18 knockdown augmented the susceptibility of resistant cells to sorafenib treatment, which could also be counteracted by Ferrostatin-1 (Fig. 3C). These results suggest that USP18 may contribute to HCC resistance by inhibiting sorafenib-induced ferroptosis.

To further investigate the role of USP18 in ferroptosis, we conducted a comprehensive analysis encompassing transmission electron microscopy (TEM) examination, BODIPY 581/591 C11 staining, FerroOrange staining, ROS staining, and malondialdehyde (MDA) assay. Consistent with our hypothesis, sorafenib treatment significantly augmented the presence of shrunken mitochondria exhibiting heavily condensed membranes and elevated levels of lipid peroxidation, Fe^2+^, and ROS in HepG2-Ctrl cells. However, these effects were comparatively less pronounced in HepG2-USP18-OE cells (Fig. 3D–H). Conversely, USP18 knockdown significantly enhanced sorafenib-induced ferroptosis in resistant cells (Supplementary Fig. S3B–E). Collectively, these findings confirm the crucial role of USP18-mediated inhibition of ferroptosis in driving sorafenib resistance.

### USP18-mediated NCOA4 deISGylation and degradation impairs sorafenib-induced ferritinophagy

**Fig. 4:**
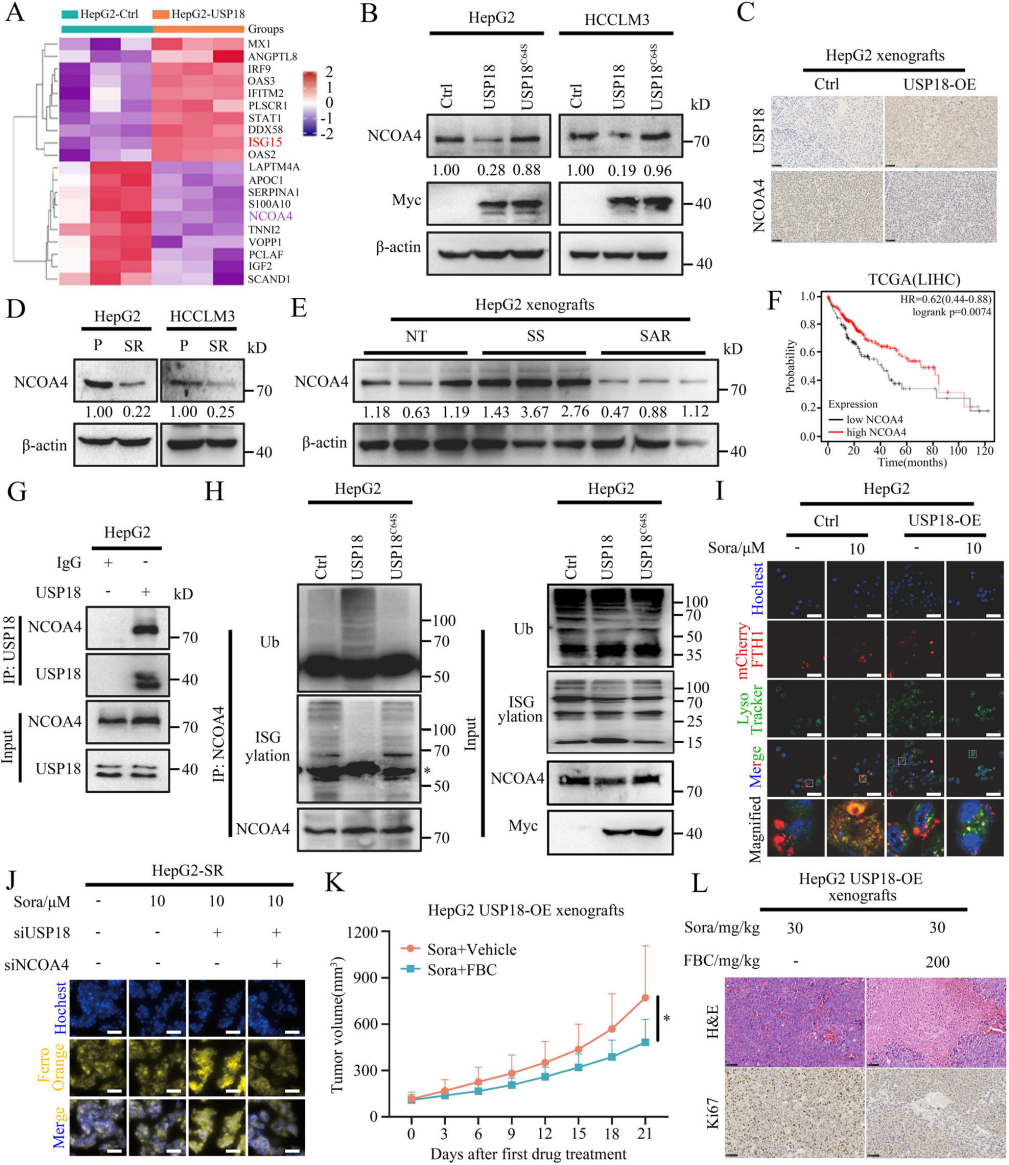
USP18-mediated NCOA4 deISGylation and degradation impairs sorafenib-induced ferritinophagy. **A** Heatmap showing the top 10 dysregulated proteins (red, upregulated proteins; purple, downregulated proteins) identified by proteomics assays. n = 3 replicates per group. **B** Western blot analysis of NCOA4 protein levels in HepG2 cells transfected with pcDNA, USP18, or USP18^C64S^ plasmid for 48 h. The intensities of bands were analyzed by Image J and normalized to the corresponding pcDNA. **C** Representative IHC staining images of USP18 and NCOA4 in HepG2-Ctrl and HepG2-USP18-OE xenografts. Scale bars, 50 μm. **D** Protein expression of NCOA4 in HCC-P and HCC-SR cells. The intensities of bands were analyzed by Image J and normalized to the corresponding HCC-P cells. **E** Protein expression of NCOA4 in the HepG2 xenografts from the indicated groups. The intensities of bands were analyzed by Image J and normalized to the mean of the corresponding NT group. **F** The Kaplan–Meier curves demonstrate the association between NCOA4 expression and overall survival among HCC patients in the TCGA cohort. **G** The HepG2 cell lysates were immunoprecipitated with anti-USP18 antibodies and blotted with anti-NCOA4 antibodies. **H** Expression of NCOA4 ubiquitination and ISGylation in anti-NCOA4 immunoprecipitation and whole-cell lysates (input) derived from HepG2 cells transfected with pcDNA, USP18, or USP18^C64S^ plasmid for 48 h and treated with MG132 (10 μM) for 4 h. *, heavy chain. **I** The effect of USP18 overexpression on NCOA4-mediated ferritinophagy. Scale bars, 5 μm. **J** FerroOrange staining. The impact of USP18 knockdown on the sorafenib-induced elevation of Fe^2+^ levels in HepG2-SR cells, with or without NCOA4 siRNA transfection. Scale bars, 5 μm. **K** The tumor growth curve in mice from the indicated groups. Tumor volume was measured every 3 days beginning from the first treatment (means ± SEM, *p < 0.05, unpaired Student’s t-test). n = 8 mice per group. **L** Representative H&E staining images and IHC images of Ki67 in excised xenografts. Scale bars, 100 μm.

To elucidate the principal downstream mediator responsible for USP18-mediated sorafenib resistance in HCC, we conducted cluster analysis on the differential protein expression based on the whole-proteome analysis of HepG2 cells with or without overexpression of USP18 (HepG2-USP18-OE vs. HepG2-Ctrl). We identified 47 commonly downregulated proteins and 31 upregulated proteins (Supplementary Fig. S4A). Among them, NCOA4, a critical regulator for ferroptosis, significantly decreased, and ISG15 upregulated in HepG2-USP18-OE cells (Fig. 4A). Western blot and IHC analysis confirmed that USP18 overexpression significantly reduced, while USP18 knockdown increased NCOA4 protein levels (Fig. 4B, C, and Supplementary Fig. S4B). Consistently, NCOA4 expression significantly decreased in both in vitro and in vivo drug-resistant models (Fig. 4D, E). Kaplan–Meier analysis of the TCGA dataset revealed a close association between decreased NCOA4 expression and unfavorable overall survival outcomes in HCC patients (Fig. 4F).

Next, we investigated how USP18 regulates NCOA4 expression. Given that the catalytic activity of USP18 relies on a conserved cysteine residue at position Cys64 [31], we generated a USP18 mutant (termed USP18^C64S^) by replacing this cysteine with serine, thus eliminating its enzymatic activity. Notably, the USP18 mutant lost its ability to regulate NCOA4 protein levels and promote sorafenib resistance (Fig. 4B and Supplementary Fig. S4C, D). Further exploration revealed that USP18 regulates NCOA4 expression in a manner dependent on ubiquitin-proteasome degradation (Supplementary Fig. S4E). Since USP18 itself lacks deubiquitinating activity [32], and previous studies have demonstrated that ISGylation can competitively bind to ubiquitination sites on substrate proteins, thereby inhibiting their ubiquitin-proteasome degradation [14, 17, 32, 33]. Based on this, we hypothesized that USP18-mediated delSGylation of NCOA4 may enhance its ubiquitination and subsequent degradation by the proteasome. Encouragingly, our findings confirmed a direct interaction between USP18 and NCOA4 (Fig. 4G). Furthermore, in Fig. 4H, overexpression of wild-type USP18 attenuated NCOA4 ISGylation, enhanced NCOA4 ubiquitination, while the USP18^C64S^ mutant did not exhibit such effects. This further supports the mechanism where USP18 promotes NCOA4 ubiquitination-proteasome degradation by catalyzing the deISGylation of NCOA4 to expose ubiquitination sites.

To further elucidate the impact of USP18 on NCOA4-mediated ferritinophagy, we utilized confocal microscopy to evaluate the co-localization of transferrin, FTH1, and lysosomes. As expected, overexpression of USP18 impeded the effectiveness of sorafenib in modulating NCOA4-mediated ferritinophagy (Fig. 4I). Consistent with this finding, ferritinophagy was significantly suppressed in sorafenib-resistant cells (Supplementary Fig. S4F). Subsequent FerroOrange staining and BODIPY 581/591 C11 staining revealed that USP18 knockdown enhanced the sensitivity of HCC-SR cells to sorafenib, which was reversed by NCOA4 knockdown (Fig. 4J and Supplementary Fig. S4G). In addition, Ferrous bis-glycinate (FBC), an orally active iron fortificant and therapeutic iron supplement, effectively restored the susceptibility and ferroptosis induction ability of sorafenib in HepG2-USP18-OE cells (Supplementary Fig. S4H, I). Furthermore, FBC enhanced the efficacy of sorafenib in suppressing tumor growth in mice bearing HepG2-USP18-OE xenografts (Fig. 4K and Supplementary Fig. S4J, K). IHC staining confirmed that the combination of sorafenib and FBC markedly decreased tumor proliferation (Fig. 4L). Taken together, these findings suggest that USP18 blocked sorafenib-induced ferroptosis via deISGylation and degradation of NCOA4, and ultimately confers HCC cells resistance to sorafenib.

### Sorafenib promotes USP18 accumulation via STING/IRF3/ISG15 axis in HCC cells

**Fig. 5:**
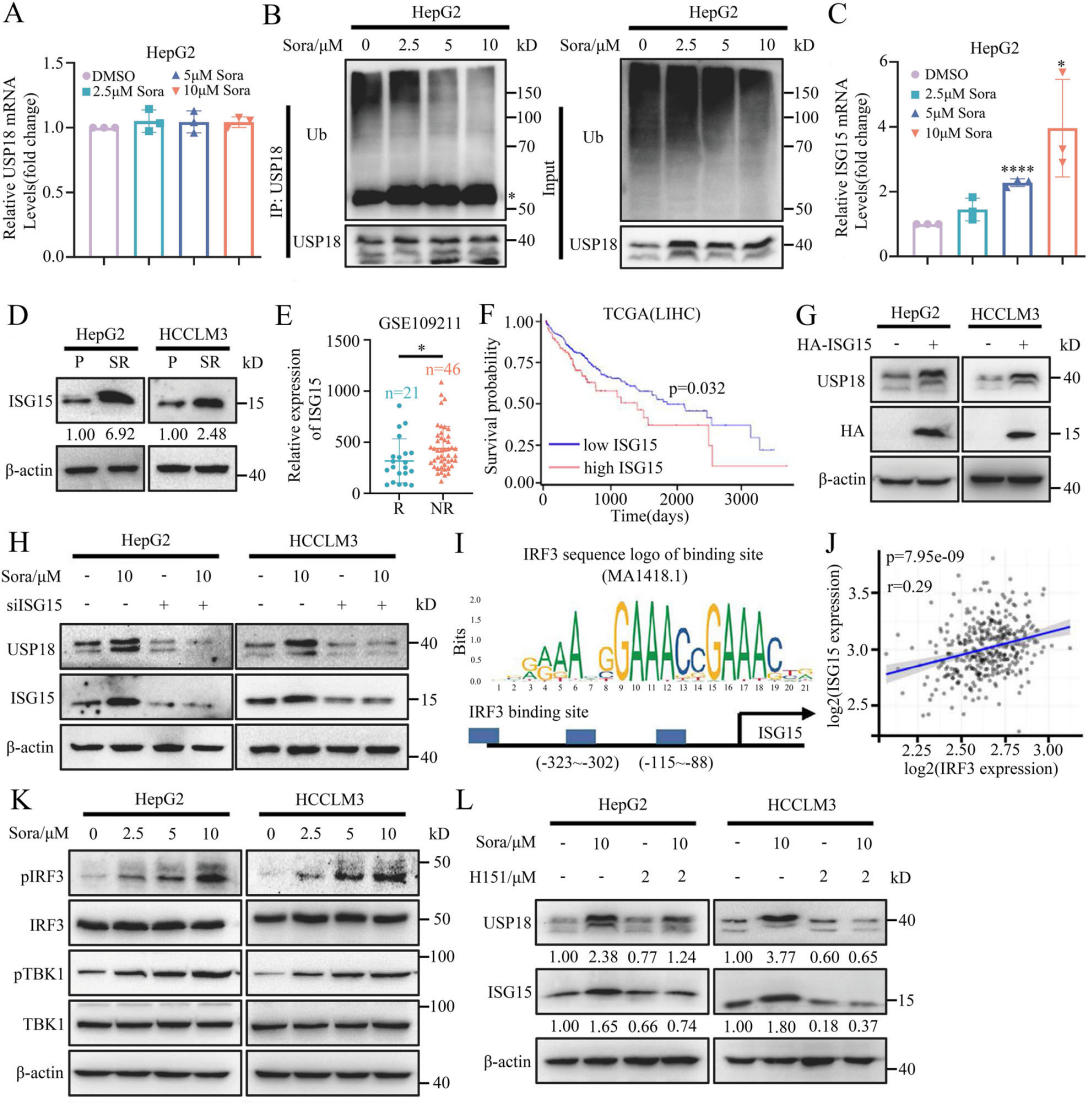
Sorafenib promotes USP18 accumulation via the STING/IRF3/ISG15 axis in HCC cells. **A** mRNA expression of USP18 in HepG2 cells treated with indicated concentrations of sorafenib for 24 h. **B** Expression of USP18 ubiquitination in anti-USP18 immunoprecipitation and whole-cell lysates (input) derived from HepG2 cells treated with indicated concentrations of sorafenib for 24 h and MG132 (10 μM) for 4 h. *, heavy chain. **C** mRNA expression of ISG15 in HepG2 cells treated with indicated concentrations of sorafenib for 24 h (means ± SEM, *p < 0.05, ****p < 0.0001, student’s t-test). **D** Protein expression of ISG15 in HCC-P and HCC-SR cells. The intensities of bands were analyzed by Image J and normalized to the corresponding HCC-P cells. **E** The ISG15 levels in HCC tissue samples of non-responders (NR) and responders (R) to sorafenib treatment were assessed using the GSE109211 dataset (means ± SEM, *p < 0.05, unpaired Student’s t-tests). **F** The Kaplan–Meier curves demonstrate the association between ISG15 expression and overall survival among HCC patients in the TCGA cohort. **G** Protein expression of USP18 in HCC cells transfected without or with HA-ISG15 plasmid for 48 h. **H** Protein expression of USP18 in HCC cells transfected without or with ISG15 siRNA for 48 h and treated with sorafenib for 24 h. **I** IRF3 binding sequence in the ISG15 promoter region was predicted with the JASPAR website. **J** Correlations between expression of IRF3 and ISG15 in HCC tissues. The r-value and p-value were calculated using Pearson correlation analysis. **K** The key protein expression of the STING signaling pathways in HCC cells treated with indicated concentrations of sorafenib for 24 h. **L** Protein expression of ISG15 and USP18 in HCC cells treated with 10 μM sorafenib and/or 2 μM H151 for 24 h. The intensities of bands were analyzed by Image J and normalized to the control cells treated with DMSO.

Another interesting question is the underlying mechanisms of USP18 accumulation in sorafenib-treated HCC cells. Firstly, we confirmed that USP18 mRNA expression showed no significant changes in sorafenib-treated HCC cells. (Fig. 5A). However, sorafenib treatment significantly decreased the ubiquitination and degradation of USP18 (Fig. 5B). It has been reported that ISG15 prevented USP18 from being degraded by the proteasome, and the depletion of intracellular ISG15 hindered the accumulation of USP18 [19, 34]. Interestingly, we observed a substantial upregulation of ISG15 mRNA and protein expression in HCC cells following sorafenib treatment (Fig. 5C and Supplementary Fig. S5A). Consistently, ISG15 expression was significantly elevated in sorafenib-resistant cells and xenografts (Fig. 5D and Supplementary Fig. 5B, C). More significantly, sorafenib non-responsive HCC patients demonstrated a notably higher expression of ISG15 in comparison to the responders (Fig. 5E), and this elevated ISG15 expression is indicative of an unfavorable prognosis among liver cancer patients (Fig. 5F). Based on these results, we speculated that elevated ISG15 may account for the USP18 upregulation. Indeed, we found that ISG15 overexpression significantly increased (Fig. 5G), while ISG15 knockdown decreased USP18 protein levels (Supplementary Fig. S5D). Furthermore, knockdown of ISG15 enhances the ubiquitination of USP18, leading to a reduction in the accumulation of USP18 in sorafenib-treated HCC cells (Fig. 5H and Supplementary Fig. S5E). These findings suggest an ISG15-dependent mechanism through which sorafenib facilitates the accumulation of USP18 protein in HCC cells.

Next, we investigated how sorafenib treatment enhances the expression of ISG15 mRNA. The transcription of ISGs mRNA is regulated by multiple signaling pathways, including the type I interferon (IFN-α/β)-JAK/STAT-ISGF3 pathway [35, 36], the type II interferon (IFN-γ)-JAK/STAT1 pathway [36, 37], the canonical cGAS-STING pathway [38], and the cGAS-STING-pIRF3-ISGs pathway [39–42]. To determine the primary signaling pathway through which sorafenib affects ISG15 mRNA expression, we first utilized the Jasper website (https://jaspar.genereg.net/) to predict potential transcription factors of ISG15, and interferon regulatory factor 3 (IRF3) exhibited the highest score (Fig. 5I and Supplementary Table S1). In addition, the Pearson correlation analysis revealed a statistically significant positive association between the expression levels of ISG15 and IRF3 in HCC samples (n_pair_ = 371, r = 0.29, p = 7.95e-09) (Fig. 5J). Additionally, we found that the STING inhibitor (H151) could reverse the expression of IRF3 downstream target genes, such as ISG15, IFNB1, and IFI44, induced by sorafenib, while the JAK inhibitor had no such effect (Supplementary Fig. S5F). These results confirm that sorafenib primarily induces the expression of ISG15 mRNA through the cGAS-STING-pIRF3-ISG15 axis.

Consistent with this, sorafenib indeed activates the STING signaling pathway in HCC cells (Fig. 5K). Moreover, H151 reverses the effect of sorafenib-induced USP18 accumulation in HCC cells and enhances the sensitivity of HCC-SR cells to sorafenib (Fig. 5L and Supplementary Fig. S5G). In summary, we demonstrate that sorafenib promotes IRF3-induced ISG15 mRNA transcription by activating the STING signaling pathway, which contributes to the stabilization and accumulation of USP18 in HCC cells.

### Identification and characterization of HYP as a USP18 inhibitor that directly targets the USP18 IBB1 domain

**Fig. 6:**
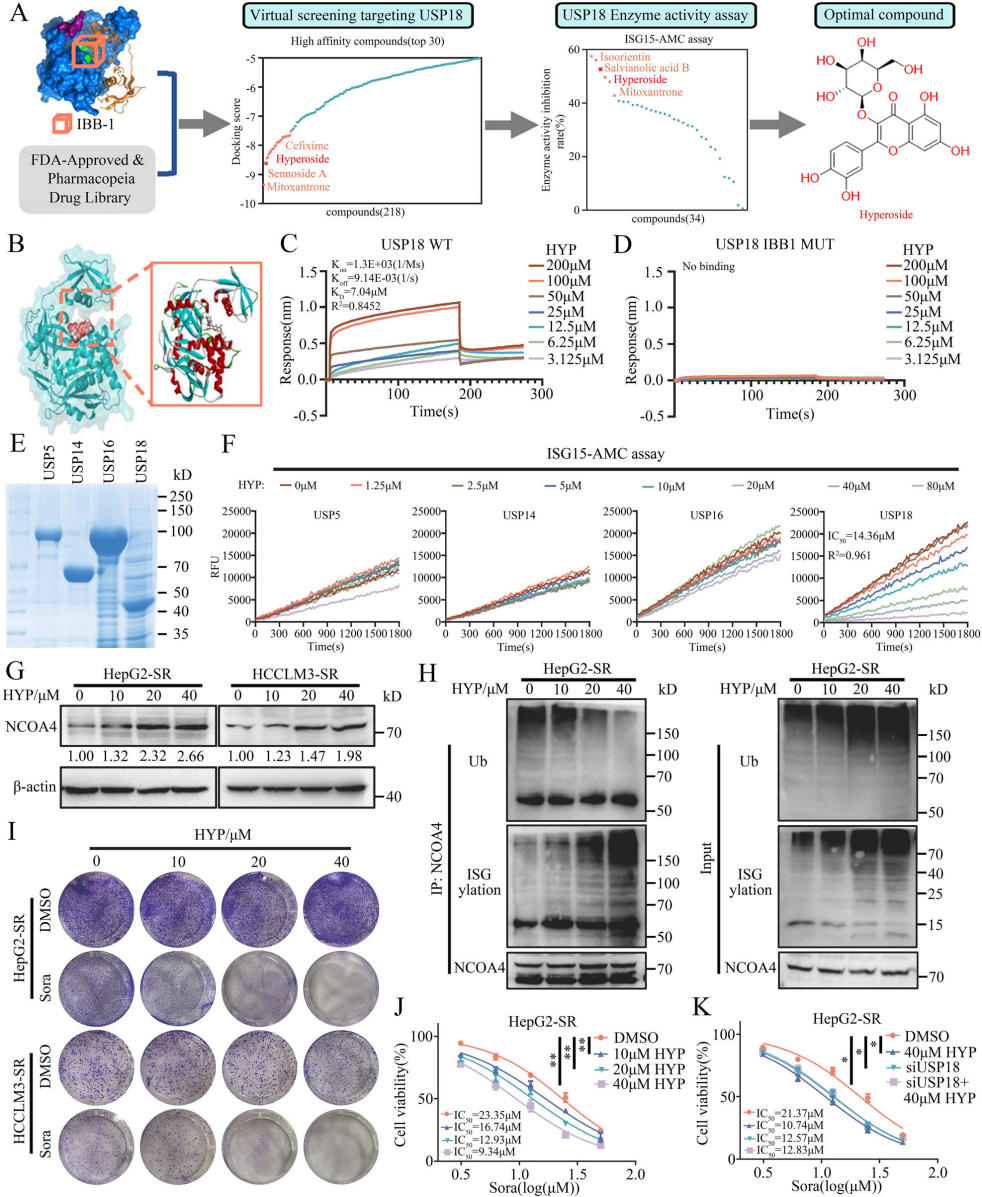
Identification and characterization of HYP as a USP18 inhibitor that directly targets the USP18 IBB1 domain. **A** The flow diagram for USP18 inhibitor screening. **B** Computational model and interactions of the USP18 IBB1 domain and HYP. **C**, **D** Biolayer interferometry (BLI) determination of the binding kinetics between HYP and USP18 WT (**C**) or USP18 IBB1 MUT (**D**). BLI response profile for HYP at different concentrations with sensor-immobilized USP18 WT or USP18 IBB1 MUT. K_D_, equilibrium dissociation constant; K_on_, association rate constant; K_off_, dissociation rate constant. **E** SDS-PAGE indicating the purification of the USP5, USP14, USP16, and USP18 proteins. **F** ISG15-AMC hydrolysis experiment. The enzymatic activity of USP5, USP14, USP16, or USP18 in the presence of increasing concentrations of HYP. **G** Protein expression of NCOA4 in HCC-SR cells treated with indicated concentrations of HYP for 24 h. The band intensities were quantified using Image J and normalized to the control cells treated with DMSO. **H** Expression of NCOA4 ubiquitination and ISGylation in anti-NCOA4 immunoprecipitation and whole-cell lysates (input) derived from HepG2-SR cells treated with indicated concentrations of HYP for 24 h and MG132 (10 μM) for 4 h. *, heavy chain. **I**, **J** Colony formation assay (**I**) and CCK-8 (**J**). The impact of HYP on the susceptibility of HepG2-SR cells toward sorafenib treatment (means ± SEM, **p < 0.01, one-way ANOVA test). **K** HepG2-SR cells were transfected with siNC or siUSP18 for 48 h and treated with sorafenib and/or 40 μM HYP for 24 h. The cell viability was measured by CCK-8 assay (means ± SEM, *p < 0.05, one-way ANOVA test).

To this end, we demonstrated that USP18 impedes sorafenib-induced ferroptosis in HCC cells through deISGylation of NCOA4, which is dependent on its enzyme activity. The IBB1 domain of the USP18 protein, consisting of residues His251, Ala138, Ser192, and Leu142, is critical for mediating direct interaction with ISG15, a prerequisite for its deISGylation activity. This domain facilitates the recruitment of ISG15-conjugated substrates, enabling USP18 to catalyze the removal of ISG15 moieties [32]. Therefore, identifying and developing USP18 inhibitors specifically targeting the IBB1 region is a novel strategy to overcome sorafenib resistance. To identify small molecules targeting the IBB1 domain of USP18, we performed a structure-based virtual screening of 3158 compounds from the FDA-Approved & Pharmacopeia Drug Library using Schrödinger software (Supplementary Fig. S6A). Based on the docking score, skeletal diversity, and molecular weight, 34 potential compounds were selected for subsequent screening (Supplementary Fig. S6B). As shown in Supplementary Fig. S6C, we conjugated 7-amino-4-methylcoumarin (AMC) to the C-terminus of ISG15 (ISG-AMC), which could be hydrolyzed by USP18, leading to the release of the fluorescent group AMC, which was indicative of USP18 activity. Therefore, we further screened the potential USP18 inhibitor based on this high-throughput screening model, and 6 compounds exhibited a significant enzyme inhibition rate exceeding 40% (Supplementary Fig. S6D). Next, we investigated the synergistic effects of compounds in combination with sorafenib in HepG2-SR cells (Supplementary Fig. S6E). Hyperoside (HYP), a flavonoid that is mainly found in *Hypericum perforatum L*., could effectively increase the sensitivity of HCC-SR cells to sorafenib (Fig. 6A).

To figure out whether HYP directly binds the IBB1 domain of USP18, we conducted a protein structure-based docking algorithm and cellular thermal shift assay (CETSA) (Fig. 6B and Supplementary Fig. S6F). In addition, the biolayer interferometry (BLI) assay further confirmed the strong interaction (K_D_ = 7.04 μM) between HYP and the IBB1 domain of USP18 (Fig. 6C, D). Furthermore, we induced and purified three reported representative deISGylating enzymes, namely USP5 [43], USP14 [44], and USP16 [45], in vitro. Furthermore, we examined the effect of HYP on the activity of these deISGylating enzymes using the ISG15-AMC hydrolysis assay (Fig. 6E, F). The results showed that HYP had the strongest inhibitory effect on USP18 enzyme activity, with only a slight inhibitory effect on USP16 enzyme activity, and almost no effect on USP5 and USP14 enzyme activities. Consistent with USP18 knockdown, treatment with HYP significantly increased the protein level of NCOA4 in HCC-SR cells (Fig. 6G). Furthermore, through CHX experiments, we confirmed that HYP prolongs the half-life of NCOA4 in a USP18-dependent manner (Supplementary Fig. S6G). Besides, we conducted a reversal experiment to confirm that the effect of HYP on increasing NCOA4 protein expression is dependent on USP18 enzymatic activity (Supplementary Fig. S6H). Additionally, further Co-IP experimental results also demonstrated that HYP treatment enhanced ISGylation and stability of NCOA4 in HCC-SR cells (Fig. 6H). Although HYP alone only exhibits slight cytotoxicity towards HCC-SR cells (Supplementary Fig. S6I), it significantly enhances the sensitivity of HCC-SR cells to sorafenib in a dose-dependent manner (Fig. 6I, J, and Supplementary Fig. S6J, K). Furthermore, we have confirmed that the role of HYP in mediating HCC-SR cell sensitivity to sorafenib is dependent on the USP18-NCOA4 signaling axis (Fig. 6K and Supplementary Fig. S7A–D). Together, these results indicate that HYP is a USP18 inhibitor and exhibits potent synergism with sorafenib in HCC-SR cells by inhibiting the deISGylation of NCOA4.

### HYP sensitizes HCC cells to both sorafenib and regorafenib treatment in the hydrodynamic transfection-induced liver cancer model

**Fig. 7:**
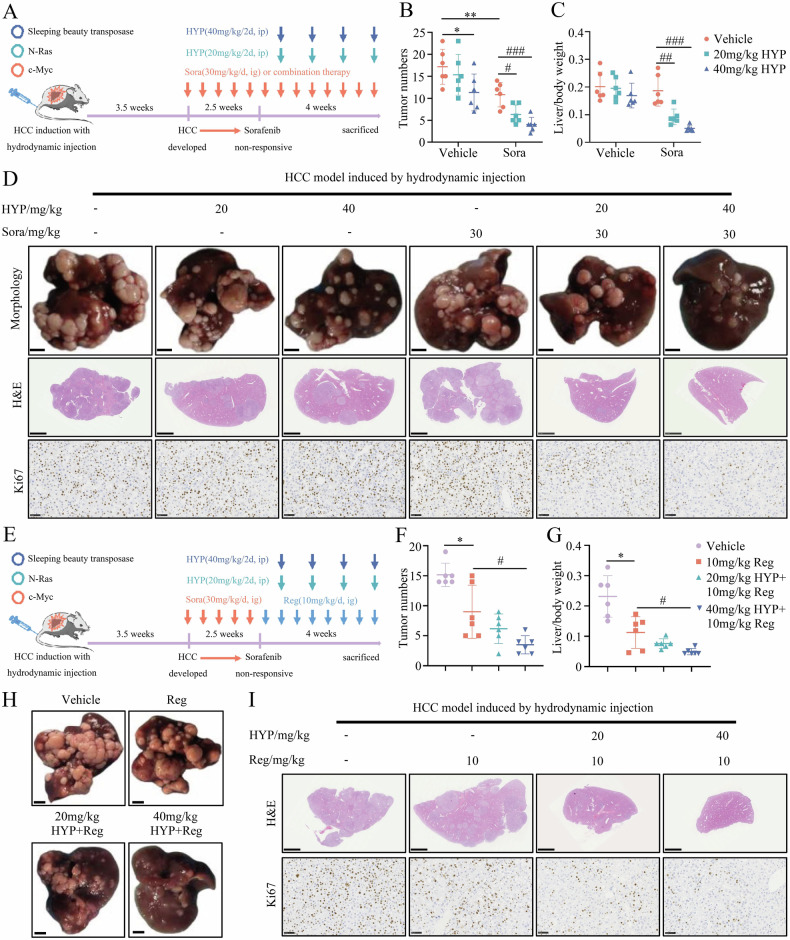
HYP sensitizes HCC cells to both sorafenib and regorafenib treatment in the hydrodynamic transfection-induced liver cancer model. **A** Schematic diagram of the treatment regimen with sorafenib (Sora) or HYP, or the combination of sorafenib (Sora) and HYP. **B**, **C** The tumor numbers in the livers (**B**) and the liver weight to body weight ratios (**C**) from mice in the indicated groups (means ± SEM, *p < 0.05, **p < 0.01 versus Vehicle group, ^#^p < 0.05, ^##^p < 0.01, and ^###^p < 0.001 versus sorafenib (Sora) group, one-way ANOVA test). **D** Representative images of liver tumors, H&E staining images, and IHC images of Ki67 in the indicated groups. n = 6 mice per group. Scale bars in the morphology, 5 mm. Scale bars in H&E, 2.5 mm. Scale bars in Ki67, 50 μm. **E** Schematic diagram of the treatment regimen with regorafenib (Reg) alone or the combination of regorafenib (Reg) and HYP. **F**, **G** The tumor numbers in the livers (**F**) and the liver weight to body weight ratios (**G**) from mice in the indicated groups (means ± SEM, *p < 0.05 versus Vehicle group, ^#^p < 0.05 versus regorafenib (Reg) group, one-way ANOVA test). **H**, **I** Representative images of liver tumors (**H**), H&E staining images, and IHC images of Ki67 (**I**) in the indicated groups. n = 6 mice per group. Scale bars in the morphology, 5 mm (**H**). Scale bars in H&E, 2.5 mm. Scale bars in Ki67, 50 μm (**I**).

Given that the RAS/MAPK and AKT/mTOR/c-Myc pathways are frequently activated in almost 50% of HCC patients [46], we performed hydrodynamic tail vein transfection of N-Ras and c-Myc proto-oncogene activation forms, which were stably integrated into the hepatocyte genome after transient expression of Sleeping Beauty transposase (SBT), for HCC induction. More importantly, this liver cancer model was induced for 3.5 weeks and then treated with sorafenib for 2.5 weeks, which could effectively simulate the clinical situation of acquired sorafenib resistance in HCC patients [47]. Finally, the groups underwent a 4-week administration protocol as illustrated in Fig. 7A. The efficacy of combination therapy was evaluated by comparing the tumor number and liver/body weight ratio with those of the single sorafenib treatment group. We found that the combination of HYP and sorafenib has shown significant potential in suppressing the growth of tumors (Fig. 7B, C, and Supplementary Fig. S7E). Consistently, Ki67 staining revealed a remarkable suppression of tumor cell proliferation in the combination treatment cohort compared to the solitary administration of sorafenib (Fig. 7D). HYP has previously been reported to exhibit antifungal activity [48], suggesting its potential to interact with multiple proteins. To demonstrate the “on-target” effect of HYP in an in vivo setting, we conducted additional assays to examine the impact of HYP on ferroptosis-related indicators (MDA and Fe^2+^) and NCOA4 protein levels in hydrodynamic-induced liver cancer tissues (Supplementary Fig. S7F–H). The results reveal that HYP can still elevate NCOA4 protein levels and enhance the ability of sorafenib to induce ferroptosis in hepatoma cells in vivo.

Regorafenib, an orally administered multi-kinase inhibitor, represents a promising second-line therapeutic option for patients with HCC who have experienced sorafenib treatment failure. However, its inherent hepatotoxicity and the occurrence of liver function abnormalities induced by sorafenib treatment in a majority of patients with HCC limit the eligibility for regorafenib treatment to approximately 30% of patients in clinical practice [49]. HYP has been reported to exhibit a favorable hepatoprotective effect [50]. Therefore, we conducted an additional evaluation to determine the potential of HYP in augmenting the therapeutic effectiveness of regorafenib for inhibiting HCC progression in vivo (Fig. 7E). Surprisingly, the combination therapy of HYP and regorafenib demonstrated a remarkable inhibition in tumor growth (Fig. 7F–H and Supplementary Fig. S7I). The decreased cell proliferation in tumors of the combination treatment group was further confirmed by Ki67 staining (Fig. 7I). In summary, our findings reveal that the inhibition of USP18 enzyme activity through HYP has the potential to sensitize and augment the effectiveness of sorafenib and regorafenib, thereby providing a promising strategy for overcoming targeted therapy resistance in HCC.

## Discussion

As the first FDA-approved molecular-targeted drug, sorafenib has been the mainstay of treatment for a decade, but the subsequent drug resistance significantly limits its therapeutic efficacy. Despite great efforts being made, the molecular mechanisms underlying sorafenib resistance remained elusive. In this study, we identified USP18 as a potential therapeutic target conferring HCC sorafenib resistance. Our findings reveal that the accumulation of USP18 blocked sorafenib-induced ferroptosis via deISGylation and degradation of NCOA4, and ultimately confers HCC cells resistance to sorafenib. The USP18 inhibitor combined with targeted therapies (sorafenib and regorafenib) significantly diminishes tumor growth in an established mouse model of multifocal HCC induced by high-pressure hydrodynamic tail vein injection of a proto-oncogene.

USP18, in addition to its demonstrated antiviral and antibacterial properties, has also been implicated in the pathogenesis and advancement of diverse malignancies [5–7, 9, 51]. USP18 was found to limit apoptotic susceptibility to IFN-α or Bortezomib in tumor cells [52]. Depletion of USP18 induces immunogenic cell death by promoting cancer cell pyroptosis [6]. Herein, we have demonstrated a gradient upregulation of USP18 in sorafenib-resistant HCC cells, xenografts, and tumor tissues obtained from patients who did not respond to sorafenib treatment. Furthermore, enhanced USP18 expression is correlated with a decline in patient survival after sorafenib treatment (Fig. 1). USP18 overexpression attenuated the susceptibility to sorafenib treatment in vivo and in vitro models. This implies that the accumulation of USP18 could be a significant determinant factor in the development of sorafenib resistance.

Increasing evidence suggests that triggering ferroptosis may represent an effective means of overcoming targeted therapy drug resistance in HCC [22, 23, 30, 53]. Through comprehensive proteomic analysis and experimental validation, we discovered that USP18 promotes drug resistance by antagonizing sorafenib-induced ferroptosis (Fig. 3). Ferroptosis is characterized by iron accumulation and lipid peroxidation [20]. Current research primarily focuses on inducing ferroptosis by targeting the GSH/GPX4 axis to modulate lipid peroxidation levels [54]. However, most GPX4 inhibitors have exhibited poor (or unclear) pharmacological properties in animal models, limiting their potential for clinical translation [53, 55]. In this study, we first found that USP18 impedes ferritinophagy by inhibiting the stability of the NCOA4 protein in HCC cells, thereby reducing intracellular Fe^2+^ levels and weakening ferroptosis-mediated drug resistance. Further investigation uncovered that this phenomenon primarily stems from USP18-mediated NCOA4 deISGylation. Unlike Lys48-linked ubiquitination, ISGylation competitively binds to ubiquitin-binding sites [14, 17, 33], inhibiting NCOA4 proteasome degradation. Although our data suggest that USP18 can directly de-ISGylate NCOA4, the observed increase in NCOA4 ubiquitination and degradation may be indirectly regulated by the synergistic action of USP18 with other E3 ligases (such as MAVS, SKP2, or CRL3KCTD10) or deubiquitinating enzymes (such as USP20) [5, 31, 56]. We will further explore the possibility of this mechanism in our subsequent studies.

The following intriguing research concerns the underlying mechanism of sorafenib’s promotion of USP18 accumulation in HCC cells. Previous research reported that IFN induced USP18 mRNA expression in tumor cells [7], and the Hedgehog (Hh) signaling pathway upregulated USP18 expression through Gli2-mediated transcriptional activation following spinal cord injury [57]. In this study, we discovered that sorafenib promotes the accumulation of USP18 in HCC cells by inducing ISG15 mRNA expression, which prevents the ubiquitination and subsequent degradation of USP18, rather than through transcriptional regulation (Fig. 1D and Fig. 5A–H). Further literature research [19, 58, 59] and experiments confirmed that sorafenib-induced ISG15 mRNA expression primarily depends on the cGAS-STING-pIRF3-ISGs signaling pathway (Fig. 5I–L and Supplementary Fig. S5H, G). Importantly, H151, a selective and covalent STING antagonist, effectively enhances the inhibitory effect of sorafenib in HCC-SR cells. This discovery broadens the understanding of the STING signaling pathway’s functions beyond enhancing antitumor immunity [60, 61], establishes a connection between the STING signaling pathway and sorafenib resistance in HCC cells, and provides a theoretical basis for the combined use of sorafenib and STING inhibitors to reverse liver cancer drug resistance.

Another important finding of the present study was the identification of HYP as an inhibitor of USP18. USP18 regulates the antiviral and antibacterial infection responses by inhibiting the IFN signaling pathway independently of its catalytic activity [8, 9]. In this study, USP18 promotes sorafenib resistance through the regulation of NCOA4 post-translational modifications in an enzyme activity-dependent manner. Hence, a targeted approach to suppress the enzymatic activity of USP18 could serve as a promising strategy to overcome drug resistance while preserving its antibacterial and antiviral efficacy. According to reports, the IBB1 domain of USP18 protein consists of His251, Ala138, Ser192, and Leu142 residues, which are crucial for mediating its direct interaction with ISG15 and are prerequisites for its deISGylation activity [32]. In this study, we demonstrated that HYP specifically targets the IBB1 domain of USP18 through a systematic screening approach. HYP showed a broad spectrum of biological activities, including anticancer, anti-inflammatory, antibacterial, antiviral, antidepressant, and organ protective effects, indicating a certain level of polypharmacology [50, 62]. Here, HYP synergistically potentiates the efficacy of targeted therapy in hydrodynamically induced liver cancer models. Therefore, this combination therapy may be a promising strategy for overcoming therapeutic resistance in HCC. However, the clinical translation of novel drugs necessitates a multi-step process [63]. Future investigations should focus on optimizing the chemical structure, profiling pharmacokinetic and toxicological characteristics, as well as conducting subsequent rigorous clinical trials for HYP.

**Fig. 8:**
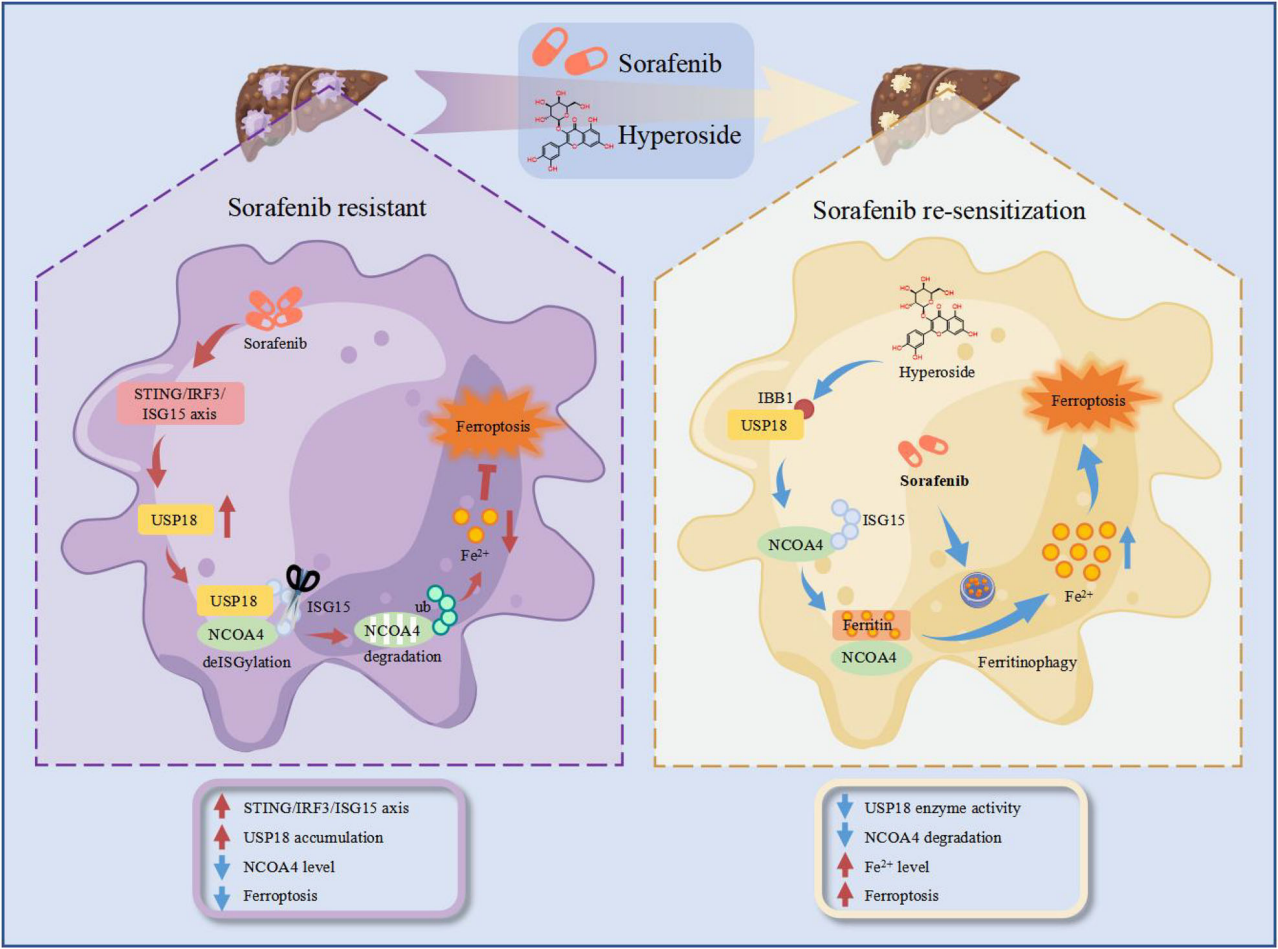
A schematic illustration of the working model. Sorafenib treatment-induced STING/IRF3/ISG15 axis activation contributes to USP18 accumulation, which promotes NCOA4 deISGylation and degradation. USP18-mediated NCOA4 deficiency impairs sorafenib-induced ferroptosis and leads to subsequent drug resistance. Hyperoside (HYP), a USP18 inhibitor, effectively enhances the sensitivity of cancer cells to sorafenib by specifically targeting the IBB1 domain and inhibiting the deISGylation process of NCOA4.

In summary, our findings have unveiled the critical role of USP18 in the development of targeted therapy resistance in HCC. Sorafenib treatment-induced STING/IRF3/ISG15 axis activation contributes to USP18 accumulation, which promotes NCOA4 deISGylation and degradation. USP18-mediated NCOA4 deficiency impairs sorafenib-induced ferroptosis and leads to subsequent drug resistance. Notably, we have identified HYP as a novel USP18 inhibitor, which sensitizes cancer cells to existing targeted therapies (sorafenib and regorafenib) by directly targeting the IBB1 domain (Fig. 8). These results uncovered a novel mechanism of acquired sorafenib resistance and offered a promising combination therapy strategy for overcoming therapeutic resistance in HCC.

## Supplementary information

Supporting information-Cell Death & Disease

uncropped Gels and Blots image(s)-Cell Death&Disease

## Data Availability

The mass spectrometry proteomics data have been deposited in the ProteomeXchange Consortium (https://proteomecentral.proteomexchange.org) via the iProX partner repository with the dataset identifier PXD047888.
