# Supplementary material for: Targeting USP18 overcomes acquired resistance in hepatocellular carcinoma by regulating NCOA4 deISGylation and ferroptosis
Source: Cell Death Dis. 2025 Jun 13;16(1):448. doi: 10.1038/s41419-025-07772-0 (PMC12166087; doi:10.1038/s41419-025-07772-0)
Supplement: Supplementary file 1 — Supporting information-Cell Death & Disease [file 41419_2025_7772_MOESM1_ESM.pdf]

Supporting Information for

Targeting USP18 overcomes acquired resistance in hepatocellular carcinoma by regulating NCOA4 deISGylation and ferroptosis

Shengtao Ye[1], Junxin Chen[1], Ying Zheng[1], Mengmeng He[1], Yanqiu Zhang[1], Yang Cheng[1], Yingrong Leng[1], Enyi Wu[1], Lingyi Kong[1,*], Hao Zhang[1,*]

Affiliations

[1] Jiangsu Key Laboratory of Bioactive Natural Product Research and State Key Laboratory of Natural Medicines, School of Traditional Chinese Pharmacy, China Pharmaceutical University, Nanjing 210009, China

* **Corresponding authors**

Lingyi Kong, State Key Laboratory of Natural Medicines and Jiangsu Key Laboratory of Bioactive Natural Product Research, School of Traditional Chinese Pharmacy, China Pharmaceutical University, 24 Tong Jia Xiang, Nanjing 210009, China. E-mail: cpu_lykong@126.com; Tel/Fax: +86-25-8327-1405

Hao Zhang, State Key Laboratory of Natural Medicines and Jiangsu Key Laboratory of Bioactive Natural Product Research, School of Traditional Chinese Pharmacy, China Pharmaceutical University, 24 Tong Jia Xiang, Nanjing 210009, China. E-mail: hiaaron@sina.cn; Tel/Fax: +86-25-8327-1405

This file includes:

Supplementary methods 3
Supplementary materials 12
Supplementary Tables 17
Supplementary Figure Legends 22

Supplementary methods

GSE data processing and survival prognosis analysis

GEO2R analysis was conducted on the GSE109211 dataset (http://www.ncbi.nlm.nih.gov/geo /geo2r/) to ascertain the differential expression of USP18 or ISG15 between HCC patients who responded favorably to sorafenib treatment and those who exhibited resistance. The UALCAN database (http://ualcan.path.uab.edu/) was employed to assess the correlation between USP18 and ISG15 expression levels and prognosis in HCC patients. Additionally, the Kaplan Meier plotter database (http://kmplot.com/analysis/) was utilized to investigate the prognostic significance of NCOA4 in HCC. Hazard ratio (HR) with 95% confidence intervals was estimated along with log-rank p-value. Statistical significance was defined as $p < 0.05$ by established criteria.

Histological analyses and immunohistochemical (IHC) staining

Xenografts or liver specimens were fixed in 10% neutral buffered formalin, followed by paraffin embedding and sectioning into 4 μm slices. Subsequently, the sections underwent deparaffinization, hydration, and staining using established protocols. Histological sections were stained with hematoxylin-eosin (H&E) to evaluate the inhibitory impact of the drug on tumors. Tissue sections were subjected to Ki67 staining to evaluate the suppressive impact of drugs on tumor proliferation. IHC was performed using paraffin sections, which were incubated with the indicated primary antibodies. The sections were scanned using a NanoZoomer 2.0 RS Pathological slide scanner (C10730-13, Hamamatsu), and the images were then digitalized.

Cell lines

HepG2 cells and HCCLM3 cells were purchased from the Cell Bank of the Type Culture Collection of the Chinese Academy of Sciences, Shanghai, China. All of the cell lines were authenticated in-house by short tandem repeat (STR) DNA profiling. To establish HepG2-SR and HCCLM3-SR cells, parental HepG2 or HCCLM3 cells were subjected to incremental concentrations of sorafenib, starting at 6 μM, until they acquired the ability to proliferate unhindered in the presence of 12 μM sorafenib. This adaptation was achieved after 24 weeks under continuous drug exposure. The HepG2-USP18-OE and HCCLM3-USP18-OE cell lines, stably over-expressing USP18, were generated by transfecting the pCMV-N-Myc-USP18 plasmid followed by G418 selection. Control cells corresponding to each line were also established. Mycoplasma contamination was assessed at least once per month, and the test results were negative.

RNA extraction, reverse transcription PCR, and quantitative real-time PCR

Total RNA was extracted from HepG2 cells, HepG2-SR cells, and HepG2-USP18-OE cells using the RNA Quick Purification Kit (ES Science, Shanghai, RN001) following the manufacturer's instructions. The quality and concentration of the RNA were assessed by measuring absorbance at 260 and 280 nm using a Thermo Scientific NanoDrop spectrophotometer. Reverse transcription of 1μg of total RNA was performed using the HiScript® II Q Select RT SuperMix for qPCR Kit (Vazyme, R232-01). Quantitative real-time PCR (qRT-PCR) was conducted on a LightCycler480 Instrument II (Roche Diagnostics Inc., Basel, BS, Switzerland) using SYBR Green qPCR Master Mix (Vazyme, Q321-02) and

corresponding primers listed in Table S2. The mRNA expression levels of target genes were normalized to GAPDH expression. The specificity of each amplicon was confirmed by analyzing its melting curve.

Protein extraction, western blot analysis and co-immunoprecipitation (Co-IP)

The total protein was isolated from liver tissues and cells were harvested and lysed on ice for 30 min using lysis buffer (Yeasen, 20118ES60) with protease inhibitor cocktail (Beyotime, P1005) and phosphatase inhibitor cocktail (Beyotime, P1045). The supernatants of lysates were collected by centrifugation at 12, 000 rpm for 10 min at 4 ℃, and the protein concentration was determined with a BCA protein assay kit (Beyotime, catalog # P0012). Supernatants were analyzed for western blot analysis or immunoprecipitation.

For western blot analysis, equal amounts of total protein were separated on 10% or 12% SDS-PAGE gels and subsequently transferred to PVDF membranes (Bio-Rad). Following a blocking step with 5% skim milk for 2 h, the membranes were incubated overnight at 4 ℃ with primary antibodies. Subsequently, the membranes were incubated with HRP-conjugated secondary antibodies for an additional 2 h. The specific antibodies used in this study are listed in the materials. Immunoreactive signals were detected using an ECL kit (170-5061; Bio-Rad; Hercules, CA) and visualized by chemiluminescence employing a ChemiDOC XRS$^+$ Molecular Imaging System (Bio-Rad). The band intensities

were quantified using Image J software and calculated based on reference bands obtained from anti-β-actin immunoblotting.

For Co-IP, the prepared cell lysates containing 1 mg total protein were precleared using 10 μL Protein A+G Agarose beads by rotating at 4 ℃ for 2-4 h. Subsequently, the indicated antibody was added to the precleared lysates, followed by the addition of 25 μL Protein A+G Agarose beads to the mixture. The tubes were then rotated at 4 ℃ for a duration of 4-6 h for each process. As a negative control, normal mouse or rabbit IgG was utilized. Following this step, the beads underwent washing with cold PBS buffer (3-5 times) and subsequently boiled with SDS loading buffer for 10 min. The immune complexes obtained were collected and subjected to western blot analysis utilizing both primary antibodies as indicated and corresponding secondary antibodies by established protocols.

Plasmid constructs

The full-length coding region of the USP18 gene was cloned into the multiple cloning site (MCS) region of the pCMV-N-Myc vector to screen for HCC cell lines that stably overexpress USP18. The full-length coding region of the ISG15 gene was inserted into the MCS region of the pCMV-C-HA vector to investigate and validate the impact of ISG15 overexpression on USP18 protein stability in HCC cells. The complete coding sequence of the USP18 gene was integrated into the MCS region of the pET28a(+) vector, and subsequently, USP18 IBB1 MUT plasmids were generated using a mutation kit to facilitate the purification of both wild-type USP18 protein and its mutant variant, USP18 IBB1 MUT protein. To

synthesize ISG15-AMC, the complete coding sequence of the ISG15 gene was inserted into the MCS region of the pTYB21 vector. The primers used for plasmid construction are listed in Table S3.

Plasmid and small-interfering RNA (siRNA) transfection

For plasmid or siRNA transfection, LipofectAMINE 2000 (Invitrogen) was employed following the manufacturer's instructions. To achieve successful overexpression or knockdown of the target gene, a mixture of 4 μg plasmid or 100 nM specific siRNA and 4 or 7.5 μL LipofectAMINE 2000 should be thoroughly combined in Opti-MEM. Following an incubation period of 8 h post-transfection, it is recommended to replace the culture medium. The assessment of overexpression or knockdown efficiency can be conducted after 48 h. The sequence of the antisense siRNA is listed in Table S4.

Cell counting kit-8 (CCK-8), 5-ethynyl-2'-deoxyuridine (EDU), and colony formation assay

For CCK-8 assay, transfected cells were cultured in a 96-well plate at a density of 8000 cells per well. Plasmid and siRNA transfection, as well as drug treatment, followed established protocols. Subsequently, each well was supplemented with 10 μL CCK-8 solution and incubated at 37 ℃ for 2-4 h. The absorbance at 450 nm was measured using Spectra-Max Plus 384 (Molecular Devices) to quantify cell viability.

For EDU assay, cells were transfected in a 96-well plate at a density of 12, 000 cells per well. Plasmid and siRNA transfection, as well as drug treatment, were performed following established protocols. Subsequently, each well was

supplemented with 20 μM EDU (Beyotime, C0071S) and incubated at 37 ℃ for 4 h. The cells were then fixed, permeabilized, and subjected to staining with Hoechst 33342 to visualize the nuclei. To determine the proportion of EDU-positive cells, cell counts were conducted in three randomly selected areas within each well.

For the colony formation assay, the transfected cells were seeded at a density of 1, 000 cells per well in a 6-well plate and treated with sorafenib and/or HYP for 24 h in complete media (2 mL). After washing with PBS, the cells were cultured in complete media for an additional 14 days. The proliferating colonies were fixed using 4% paraformaldehyde and stained with crystal violet.

Transmission electron microscope assay

Cells were collected and fixed with 2.5% glutaraldehyde, followed by post-fixation in 2% tetroxide and dehydration through a series of ethanol gradients. Subsequently, the samples were embedded in epoxy resin, sectioned into thin slices, and placed onto nickel grids. High-resolution images were acquired using a Hitachi-7500 transmission electron microscope (Hitachi, Tokyo, Japan).

Determination of lipid peroxidation

BODIPY 581/591 C11 (Thermo Fisher) was employed for the detection of lipid peroxidation by the manufacturer's instructions. The quantification of malondialdehyde (MDA) content was performed using respective biochemical assay kits (Beyotime, S0131S).

Measurement of total Fe^{2+} levels in HCC cells

The level of Fe^{2+} was determined using FerroOrange (1 μM, MKBio, MX4559) by the manufacturer's instructions. Briefly, cells were transfected in a 96-well plate at a density of 12, 000 cells per well following established protocols for plasmid and siRNA transfection as well as drug treatment. Subsequently, each well was incubated with 1 μM FerroOrange at 37 ℃ for 30 min and then stained with Hoechst 33342 to visualize the nuclei. Fluorescence intensity was analyzed under an ImageXpress Micro Confocal Platform (Molecular Devices) equipped with a 60x objective lens. (Abs_{max}=542 nm, FL_{max}=572 nm).

Measurement of intracellular reactive oxygen species (ROS) generation

Intracellular levels of ROS were quantified using the ROS assay kit (Beyotime, China) through the conversion of a DCFH-DA fluorescence probe. Briefly, HCC cells were transfected with plasmid or siRNA for 36 h and subsequently treated with DMSO or sorafenib for 24 h. Following this, cells were incubated at a final concentration of 10 μM DCFH-DA fluorescence probe for 20 min at 37 ℃ and stained with Hoechst 33342 to visualize the nuclei. Finally, fluorescence intensity was analyzed under an ImageXpress Micro Confocal Platform (Molecular Devices) equipped with a 60x objective lens.

Cellular thermal shift assay (CETSA)

Briefly, HepG2-USP18-OE cells were seeded in 10 cm culture dishes and cultured until reaching a confluence of 70-80%. Subsequently, the cells were treated with 40 μM HYP or DMSO for 4 h. The cells were then collected, pelleted, and washed with PBS before being resuspended to a density of $5×10^6$ cells/mL

in PBS supplemented with protease inhibitor. Following this, 100 μL of each cell suspension was dispensed into PCR tubes and subjected to thermal cycling at temperatures ranging from 42-56 ℃ for 3 min. The cells were immediately lysed by freeze-thawing in liquid nitrogen after heating. To clarify the cell lysates, centrifugation was performed at a speed of 20, 000 g for 20 min at a temperature of 4 ℃. Finally, the supernatants obtained were analyzed through Western blot.

ISG15-AMC hydrolysis assay

ISG15-AMC hydrolysis assay was performed to determine the inhibitory effect of compounds on USP18 enzyme activity. Excess-free AMC in the samples was removed by dialyzing in ISG15-AMC reaction buffer (50 mM Tris, 250 mM NaCl, pH 7.5). Candidate compounds were accurately weighed and dissolved in DMSO to prepare a 1 mM solution for subsequent use. Each reaction consisted of 0.5 μL compound sample, 2.5 μL prepared USP18 enzyme solution, and 19.5 μL reaction buffer (50 mM Tris-HCl, 1 mM EDTA, 1 mM ATP, 5 mM $MgCl_2$, 1 mM DTT, 1 mg/mL Ovalbumin, pH 7.5), which was incubated at a temperature of 37 ℃ for 5 min before adding 2.5 μL ISG15-AMC. A positive control well (replacing the compound sample with 0.5 μL DMSO) and a blank substrate well (replacing the USP18 with an equal volume of 2% DMSO) were set up simultaneously. The reaction progress was monitored using SpectraMax Paradigm Multi-Mode Detection Platform (Molecular Devices) equipped with an optical module operating at an excitation wavelength of 380 nm and emission wavelength of 460 nm. Measurements were taken every 20 s for a total duration

of 10 min. The inhibition rate of USP18 enzyme activity was calculated based on the derived reaction rate obtained from enzymatic activity measurements.

Supplementary materials

Reagent or Resource	Source	Identifier
**Antibodies**		
anti-USP18, WB, dil:1/1000; IP, dil: 1/50	Cell Signaling Technology	Cat# 4813; RRID: AB_10614342
anti-ubiquitin, WB, dil: 1/1000	Cell Signaling Technology	Cat# 20326; RRID: AB_3064918
anti-Myc tag, WB, dil: 1/1000	Cell Signaling Technology	Cat# 2276; RRID: AB_331783
anti-TBK1, WB, dil: 1/1000	Cell Signaling Technology	Cat# 3504; RRID: AB_2255663
anti-pTBK1, WB, dil: 1/1000	Cell Signaling Technology	Cat# 5483; RRID: AB_10693472
anti-IRF3, WB, dil: 1/1000	Cell Signaling Technology	Cat# 4302; RRID: AB_1904036
anti-pIRF3, WB, dil: 1/1000	Cell Signaling Technology	Cat# 4947; RRID: AB_823547
anti-NCOA4, WB, dil:1/1000; IP, dil: 1/100	Abcam	Cat# ab86707; RRID: AB_1925236
anti-USP18, IHC, dil:1/100	Abclonal	Cat# A16739; RRID: AB_2772822
anti-NCOA4, IHC, dil: 1/100	Aifang Biological	Cat# AF04009;
anti-ISG15, WB, dil:1/1000	Proteintech	Cat# 15981-1-AP; RRID: AB_2126302
anti-β-Actin, WB, dil: 1/1000	Proteintech	Cat# 20536-1-AP; RRID: AB_10700003
anti-HA tag, WB, dil: 1/1000	YEASEN	Cat# 30702ES60; RRID: AB_2920545
anti-normal rabbit IgG, IP, 2μg	Cell Signaling Technology	Cat# 2729; RRID: AB_1031062
anti-mouse IgG control, IP, 2μg	Cell Signaling Technology	Cat# 5415; RRID: AB_10829607
Peroxidase AffiniPure Goat Anti-Mouse IgG(H+L), WB, dil: 1/10000	YEASEN	Cat# 33201ES60; RRID: AB_10015289

Continued		
**Reagent or Resource**	**Source**	**Identifier**
Peroxidase AffiniPure Goat Anti-Rabbit IgG(H+L), WB, dil: 1/10000	YEASEN	Cat# 34201ES60; RRID: AB_10015282
**Experimental models: Cell lines**		
HepG2	Cell bank of CAS	N/A
HCCLM3	Cell bank of CAS	N/A
HepG2-SR	This paper	N/A
HCCLM3-SR	This paper	N/A
HepG2-USP18-OE	This paper	N/A
HCCLM3-USP18-OE	This paper	N/A
Competent cells: DH5α	GENERAL BIOL	N/A
Competent cells: BL21	GENERAL BIOL	N/A
**Experimental models: Organisms/strains**		
BALB/c Nude mice	Vital River Laboratory	N/A
C57BL/6J mouse	Vital River Laboratory	N/A
**Biological samples**		
HCC tissues microarray	LD BIO	Cat# LVC1609
**Plasmid**		
N-Ras	Addgene	Cat# 14723
C-myc	Addgene	Cat# 102625
Sleeping Beauty transposase	Addgene	Cat# 34879
pET-28a(+)	Addgene	Cat# 69864
pCMV-C-HA	Beyotime	Cat# D2639
pCMV-N-Myc	Beyotime	Cat# D2756
pCMV-N-mCherry	Beyotime	Cat# D2711
pTYB21	NEB	Cat# N6709
pTYB21-ISG15	This paper	N/A
pCMV-C-HA-ISG15	This paper	N/A
pET-28a(+)-USP18	This paper	N/A
pCMV-N-Myc-USP18	This paper	N/A
pCMV-N-mCherry-FTH1	This paper	N/A
pET-28a(+)-USP18 IBB1 Mut	This paper	N/A

Continued		
**REAGENT or RESOURCE**	**SOURCE**	**IDENTIFIER**
**Oligonucleotides**		
Primer for qPCR, see Table S2	This paper	N/A
Primer for PCR, see Table S3	This paper	N/A
Primer for siRNA, see Table S4	This paper	N/A
**Chemicals, peptides, recombinant proteins, and critical commercial assays**		
DAPI Staining Solution	Beyotime	Cat# C1006
PMSF	Beyotime	Cat# ST506
Penicillin-Streptomycin	Beyotime	Cat# C0222
PBS	Beyotime	Cat# C0221
Crystal Violet	Beyotime	Cat#C0121
Protease inhibitor cocktail	Beyotime	Cat# P1005
Phosphatase inhibitor	Beyotime	Cat# P1081
MG-132	Beyotime	Cat# S1748
BCA protein assay kit	Beyotime	Cat# P0010
His-tag purification resin kit	Beyotime	Cat# P2226
1M Tris-HCl, pH6.8	Beyotime	Cat# ST768
1M Tris-HCl, Ph8.8	Beyotime	Cat# ST788
TEMED	Beyotime	Cat# ST728
30% ACR-Bis	Beyotime	Cat# ST003
10% APS	Beyotime	Cat# ST005
EdU Cell Proliferation Kit with Alexa Fluor 488	Beyotime	Cat# C0071
Coomassie Blue Fast Staining Solution	Beyotime	Cat# P0017
Reactive Oxygen Species Assay Kit	Beyotime	Cat# S0033
Hochest 33342	Beyotime	Cat# C1029
DAPI Staining Solution	Beyotime	Cat# C1006
DTT	Beyotime	Cat# ST043
MDA assay kit	Beyotime	Cat# S0131S
RNA Quick Purification Kit	ES Science	Cat# RN001

Continued		
**REAGENT or RESOURCE**	**SOURCE**	**IDENTIFIER**
Trypsin	Gibco	Cat# 27250-018
Fetal Bovine Serum (FBS)	Gibco	Cat# 16140071
DMEM	Gibco	Cat# 11885084
RPMI 1640	Gibco	Cat# 11875101
Lipofectamine 2000 Transfection Reagent	Invitrogen	Cat# 11668019
BODIPY™ 581/591 C11	Invitrogen	Cat# D3861
Protein A/G Magnetic Beads	MedChemExpress	Cat# HY-K0202
PEG300	MedChemExpress	Cat# HY-Y0873
Cell counting kit-8 (CCK-8)	MedChemExpress	Cat# HY-K0301
sorafenib	MedChemExpress	Cat# HY-10201
hyperoside	MedChemExpress	Cat# HY-N0452
Ferrostatin-1	MedChemExpress	Cat# HY-100579
ZVAD-FMK	MedChemExpress	Cat# HY-16658B
Necrostatin-1	MedChemExpress	Cat# HY-15760
H151	MedChemExpress	Cat# HY-112693
Ferrous bis-glycinate	MedChemExpress	Cat# HY-130078
EcoR Ⅰ	NEB	Cat# R0101
BamH Ⅰ	NEB	Cat# R0136
Hind Ⅲ	NEB	Cat# R0104
IPTG	Beyotime	Cat# ST098
Chitin Resin	Sangon Biotech	Cat# C500097-0005
Agarose	Sangon Biotech	Cat# A620014
Octet® Ni-NTA Biosensors	SARTORIUS	Cat# 18-5101
Glutaraldehyde Fixed Solution	Servicebio	Cat# G1102
FerroOrange (Fe^{2+} indicator)	Shanghai Maokang	Cat# MX4559
MesNa	Shanghai yuanye	Cat# S16035
Gly-AMC	Shanghai yuanye	Cat# Y70814
NHS	Shanghai yuanye	Cat# S30615
Triton X-100	Sigma-Aldrich	Cat# T8787
ECL kit	UU Bio	Cat# U10012

Continued		
**REAGENT or RESOURCE**	**SOURCE**	**IDENTIFIER**
FDA-Approved & Pharmacopeia Drug Library	TargetMol	Cat# L1010
HiScript III RT SuperMix for qPCR	Vazyme	Cat#R323-01
ChamQ SYBR qPCR Master Mix	Vazyme	Cat#Q341-02
HiScript II Q RT SuperMix for qPCR	Vazyme	Cat# R222
ChamQ SYBR qPCR Master Mix(Without ROX)	Vazyme	Cat# Q321
ClonExpress II One-Step Cloning Kit	Vazyme	Cat# C112
Mut Express MultiS Fast Mutagenesis Kit V2	Vazyme	Cat# 215
2 × Phanta Max Master Mix (Dye Plus)	Vazyme	Cat# P525
DNA Marker DL2000	Vazyme	Cat# MD101-01
WB/IP lysis buffer	YEASEN	Cat# 20118ES60
LysoSensor™ Green DND-189	YEASEN	Cat# 40767ES50
**Software and algorithms**		
Prism 8.0	GraphPad	N/A
ImageJ	Open source	N/A
Image Lab software	BIO-RAD	N/A
NDP. VIEW 2.3.1	Hamamatsu Photonic K.K.	N/A

Supplementary Tables

Table S1. The information on the top five potential transcription factors of ISG15, Related to Fig. 5I.

Matrix ID	Name	Score	Relative score	Sequence ID	Start
**MA1418.1**	**IRF3**	**30.260056**	**0.988759**	**Hg38_knownGene_ENST00000649529.1**	**1886**
MA1596.1	ZNF460	25.988571	0.990282	Hg38_knownGene_ENST00000649529.1	1893
MA0517.1	STAT1::STAT2	21.051506	0.986147	Hg38_knownGene_ENST00000649529.1	827
MA0671.1	NFIX	9.016852	0.957786	Hg38_knownGene_ENST00000649529.1	641
MA0161.1	NFIC	8.52041	0.960578	Hg38_knownGene_ENST00000649529.1	642

Table S2. Primers for qPCR, Related to Fig. 5, Fig. S5

Genes	Forward primer (5'-3')	Reverse primer (5'-3')
**human**		
IFNB1	CAGCATCTGCTGGTTGAAGA	CATTACCTGAAGGCCAAGGA
IFI44	GGTGGGCACTAATACAACTGG	CACACAGAATAAACGGCAGGTA
USP18	CCTGAGGCAAATCTGTCAGTC	CGAACACCTGAATCAAGGAGTTA
ISG15	CGCAGATCACCCAGAAGATCG	TTCGTCGCATTTGTCCACCA
GAPDH	CTGGGCTACACTGAGCACC	AAGTGGTCGTTGAGGGCAATG

Table S3. Primers for PCR, Related to construction of plasmid

Genes	Forward primer (5'-3')	Reverse primer (5'-3')
**human**		
pTYB21-ISG15	CGCGATATCGTCGACGGATCCATGGGCTGGGACCTGACGGT	TTAATTACCTGCAGGGAATTCGCTCCGCCCGCCAGGCTCTG
pCMV-C-HA-ISG15	CGCTCTAGCCCGGGCGGATCCATGGGCTGGGACCTGACGGT	ATCGAATTCCTGCAGAAGCTTGCTCCGCCCGCCAGGCTCTG
pET-28a(+)-USP18	CAGCAAATGGGTCGCGGATCCATGAGCAAGGCGTTTGGGCT	TTGTCGACGGAGCTCGAATTCGCACTCCATCTTCATGTAAA
pET-28a(+)-USP5	CAGCAAATGGGTCGCGGATCCATGGCGGAGCTGAGTGAGGA	TTGTCGACGGAGCTCGAATTCGCTGGCCACTCTCTGGTAGA
pET-28a(+)-USP14	CAGCAAATGGGTCGCGGATCCATGCCGCTCTACTCCGTTAC	TTGTCGACGGAGCTCGAATTCCTGTTCACTTTCCTCTTCCA
pET-28a(+)-USP16	CAGCAAATGGGTCGCGGATCCATGGGAAAGAAACGGACAAA	TTGTCGACGGAGCTCGAATTCCAGTATTCTCTCATAAAATA
pCMV-N-Myc-USP18	GATCTGAGCCCGGGCGGATCCATGAGCAAGGCGTTTGGGCT	TCTGTCGACGATATCGAATTCGCACTCCATCTTCATGTAAA
pET-28a(+)-USP18 ALA141 A/Q	TCAACAACTGTACCGCAAACTCTGGAACCTGATTAAGGACCA	TGCGGTACAGTTGTTGAGCATCATGTTGGACAAACAAGGGC
pET-28a(+)-USP18 SER197 S/Q	CCCACTTCAACTTTTTGATGTGGACTCAAAGCCCCTGAAG	CAAAAAGTTGAAGTGGGAGGGTGAGCATGCTGCTGTTTCT
pET-28a(+)-USP18 HIS255 H/Q	GACAATCCAACTCATGCGATTCTCCATCAGGAATTCACAGACG	GCATGAGTTGGATTGTCAGGGTCTGGGGCAAATGGGTC

pCMV-N-mCherry-FTH1	GACGAGCTGTACAAGGGATCCATGACGACCGCGTCCACCTC	TCTGTCGACGATATCGAATTCGCTTTCATTATCACTGTCTC

Table S4. sequence of siRNA

Sequence for siRNA(5'-3')	
USP18 siRNA-1	CCAGGGAGTTATCAAGCAA
USP18 siRNA-2	CATCCGGAATGCTGTGGAT
ISG15 siRNA-1	GCACCGUGUUCAUGAAUCUUU
ISG15 siRNA-2	GCAACGAAUUCCAGGUGUC
NCOA4 siRNA-1	CCCAGGAAGTATTACTTAATT
NCOA4 siRNA-2	GCTGGCAAACAGAAGTTTAAA

Supplementary Figure Legends

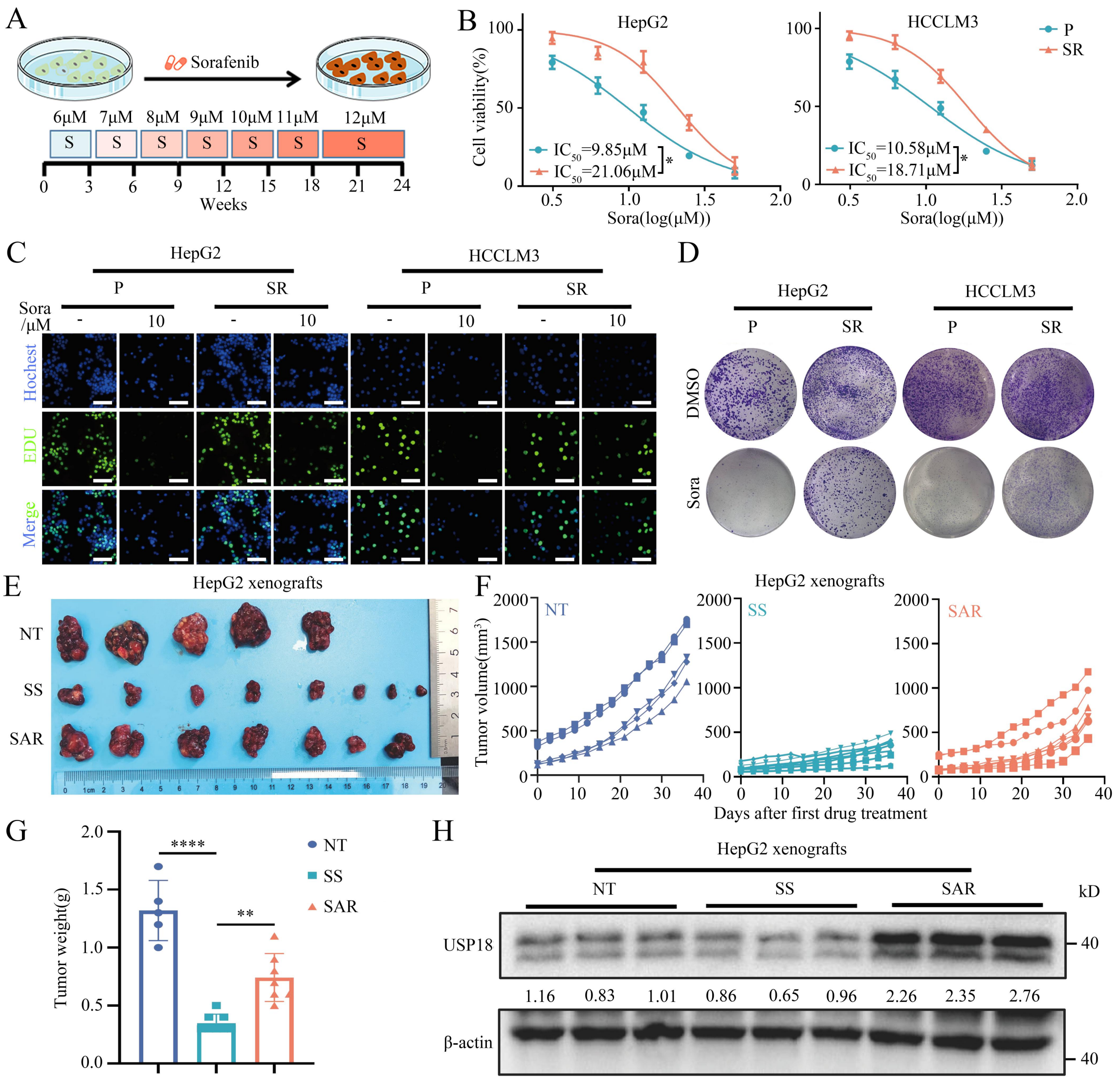

Figure S1. Construction of HCC-SR model in both *in vivo* and *in vitro* settings. (A) Schematic diagram of the establishment of HCC-SR model *in vitro*. **(B-D)** CCK-8 (B), EDU (C), and colony formation assay (D). The successful establishment of two HCC-SR cell lines (means ± SEM, *p < 0.05, paired student's t-test). **(E)** Representative pictures of subcutaneous HepG2 xenografts from the indicated groups (n ≥ 5 mice per group). **(F)** The tumor growth curve of

HepG2 cells in nude mice from different groups. Tumor volume was measured every 3 days beginning from the first treatment. **(G)** The tumor weights from the indicated groups (means ± SEM, $**p < 0.01$, $****p < 0.0001$, one-way ANOVA test). $n \geq 5$ mice per group. **(H)** Protein expression of USP18 in the HepG2 xenografts from the indicated groups. The intensities of bands were analyzed by Image J and normalized to the mean of the corresponding NT group.

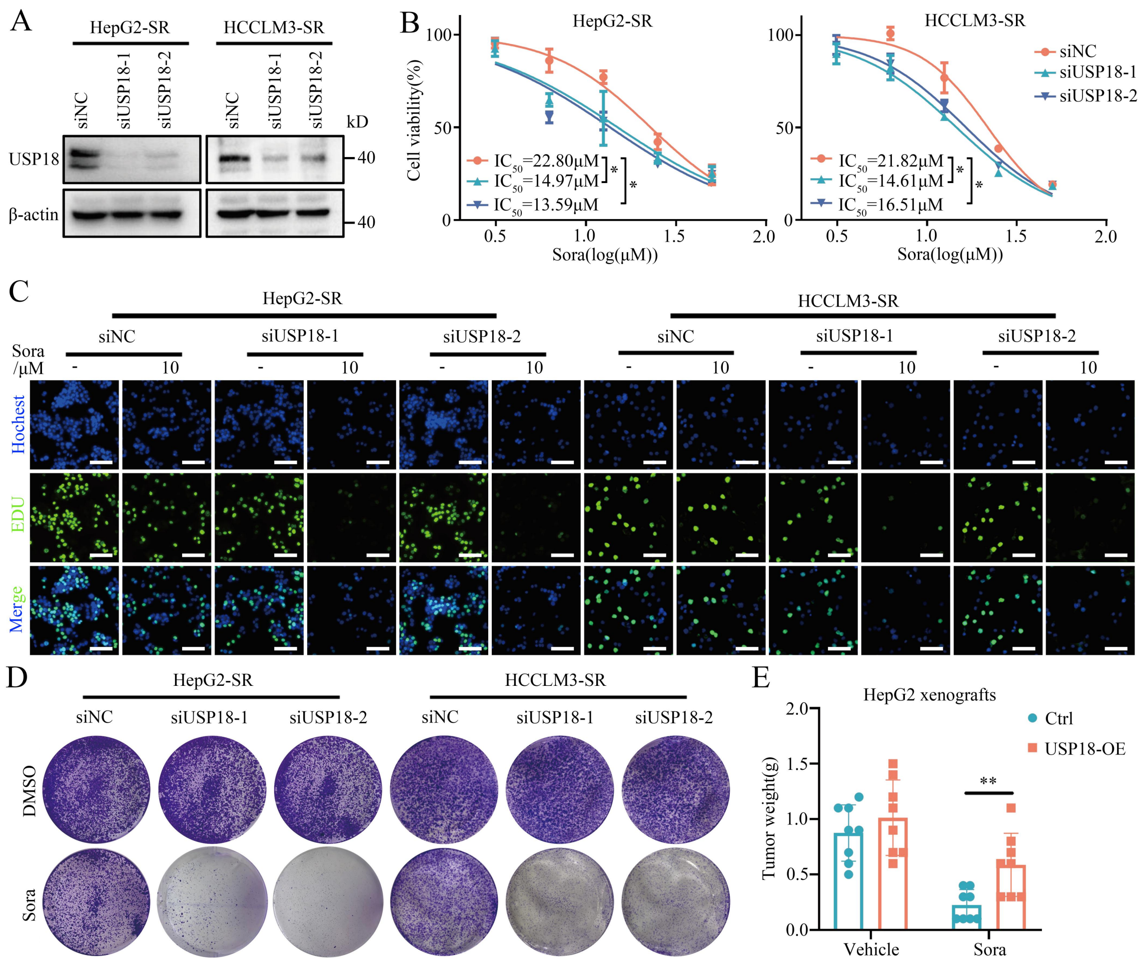

Figure S2. USP18 knockdown sensitizes HCC-SR cells to sorafenib treatment. (A) Protein expression of USP18 in HCC-SR cell lines after transfection of USP18 siRNAs for 48 h. **(B-D)** CCK-8 (B), EDU (C), and clone formation assay (D). The impact of USP18 knockdown on the susceptibility of HCC-SR cells toward sorafenib treatment (means ± SEM, *$p < 0.05$, one-way ANOVA test). Scale bars, 5 μm (D). **(E)** The tumor weights from the indicated groups (means ± SEM, **$p < 0.01$, unpaired student's t-test). n = 8 mice per group.

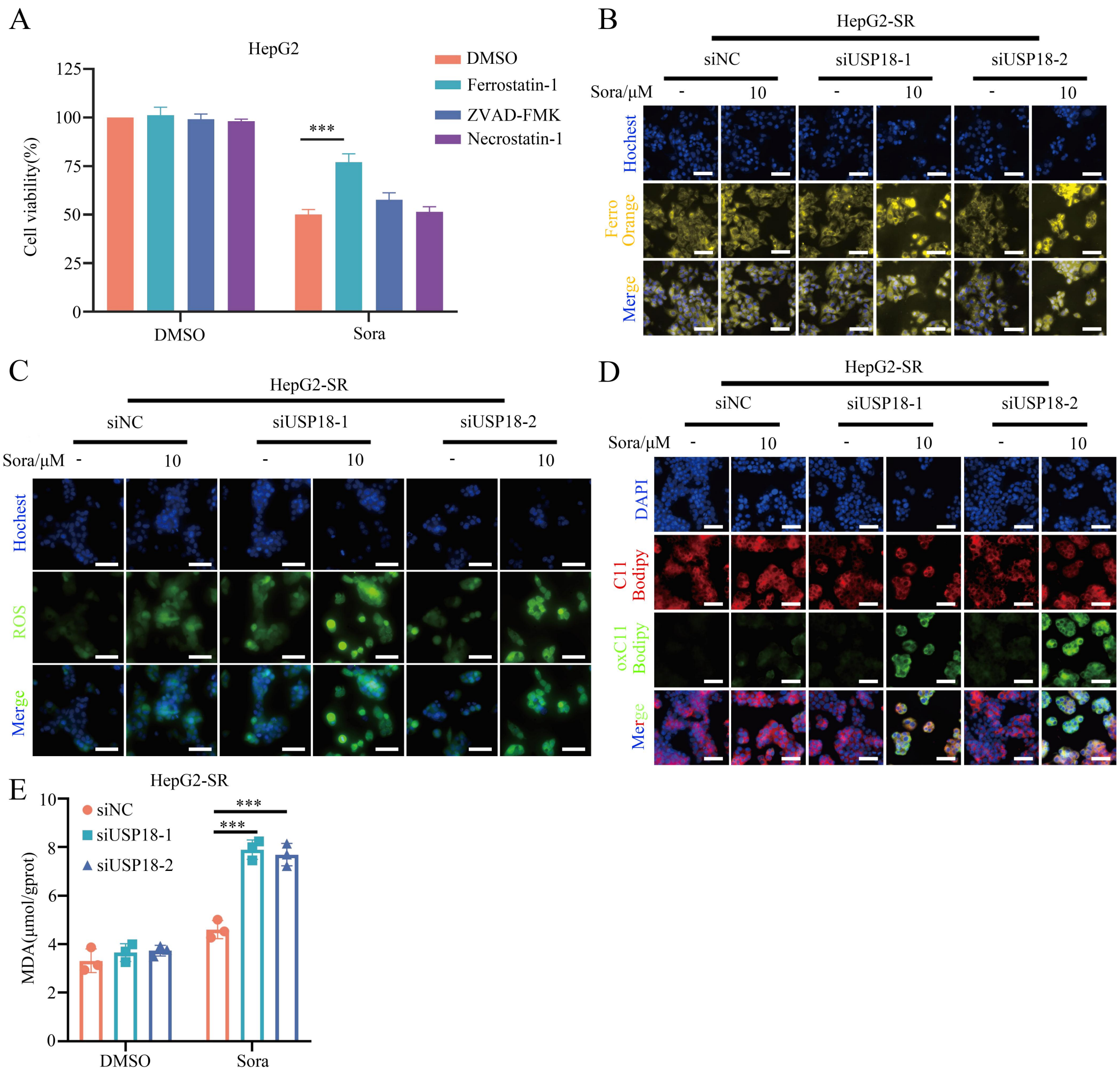

Figure S3. USP18 knockdown enhances the efficacy of sorafenib-induced ferroptosis in HCC-SR cells. (A) CCK-8 assay. HepG2 cells were treated with DMSO or 10 μM sorafenib in the absence or presence of ferrostatin-1(2 μM), Necrosulfonamide (0.5 μM), and ZVAD-FMK (10 μM) for 24 h (means ± SEM, ***$p < 0.001$, one-way ANOVA test). **(B)** FerroOrange staining. The impact of USP18 knockdown on the sorafenib-induced elevation of Fe^{2+} levels in HepG2-SR cells. Scale bars, 5 μm. **(C)** ROS staining. The effect of USP18 knockdown

on the generation of ROS induced by sorafenib in HepG2-SR cells. Scale bars, 5 μm. **(D, E)** BODIPY 581/591 C11 staining (D) and MDA assay (E). The impact of USP18 knockdown on sorafenib-induced lipid peroxidation (mean ± SEM, ***$p < 0.001$, one-way ANOVA test). Scale bars, 5 μm.

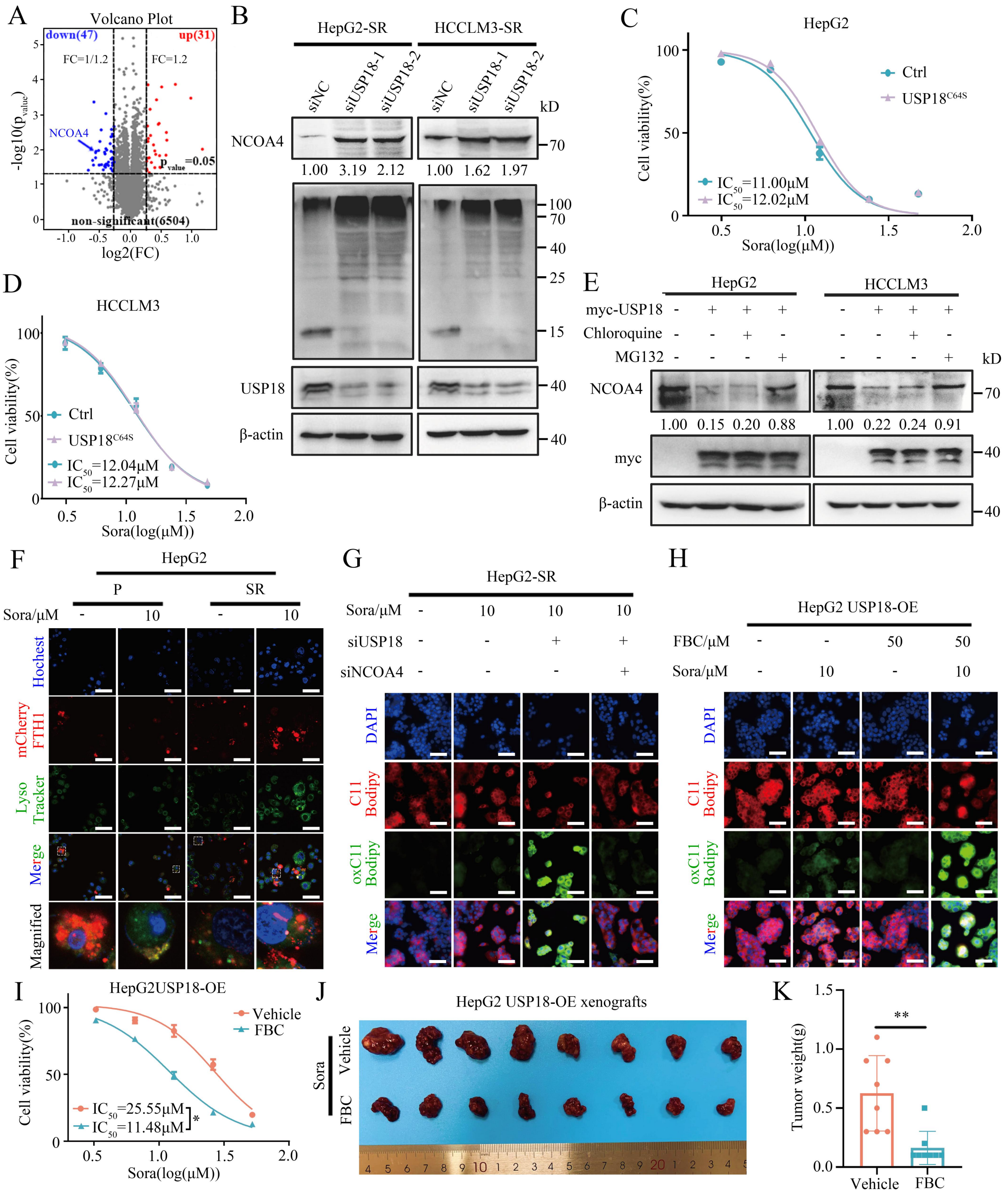

Figure S4. USP18 inhibits the sorafenib-induced ferritinophagy by decreasing NCOA4. (A) Volcano plot showing dysregulated proteins (red, up-regulated proteins; blue, down-regulated proteins) identified by proteomics

assays. NCOA4 was denoted by blue arrows. **(B)** Western blot analysis of NCOA4 and ISG15 protein levels in HCC-SR cells transfected with or without USP18 siRNAs for 48 h. The intensities of bands were analyzed by Image J and normalized to the group transfected with siNC. **(C, D)** CCK-8 assay. The impact of USP18^{C64S} overexpression on the susceptibility of HCC cells toward sorafenib treatment. **(E)** HepG2 and HCCLM3 cells were transfected with myc-USP18 plasmid or control plasmid and were cultured for 48 h before being further incubated with MG132 (10 μM) for 4 h or chloroquine (25 μM) for 6 h. The NCOA4 protein levels of the transfected cells were detected by western blot. **(F)** NCOA4-mediated ferritinophagy in HepG2-P and HepG2-SR cells was assessed by examining the co-localization of transferrin FTH1 with lysosomes. Scale bars, 5 μm. **(G)** BODIPY 581/591 C11 staining. The impact of USP18 knockdown on the sorafenib-induced elevation of lipid peroxidation in HepG2-SR cells, with or without NCOA4 siRNA transfection. **(H)** BODIPY 581/591 C11 staining. The combined treatment of FBC and sorafenib on the sorafenib-induced elevation of lipid peroxidation in HepG2 USP18-OE cells. Scale bars, 5 μm. **(I)** CCK-8 assay. The influence of FBC on the susceptibility of HepG2 USP18-OE cells towards sorafenib treatment (means ± SEM, $*p < 0.05$, paired student's t-test). **(J)** Representative pictures of subcutaneous HepG2-USP18-OE xenografts from the indicated groups (n = 8 mice per group). **(K)** The tumor weights from the indicated groups (means ± SEM, $**p < 0.01$, unpaired student's t-test). n = 8 mice per group.

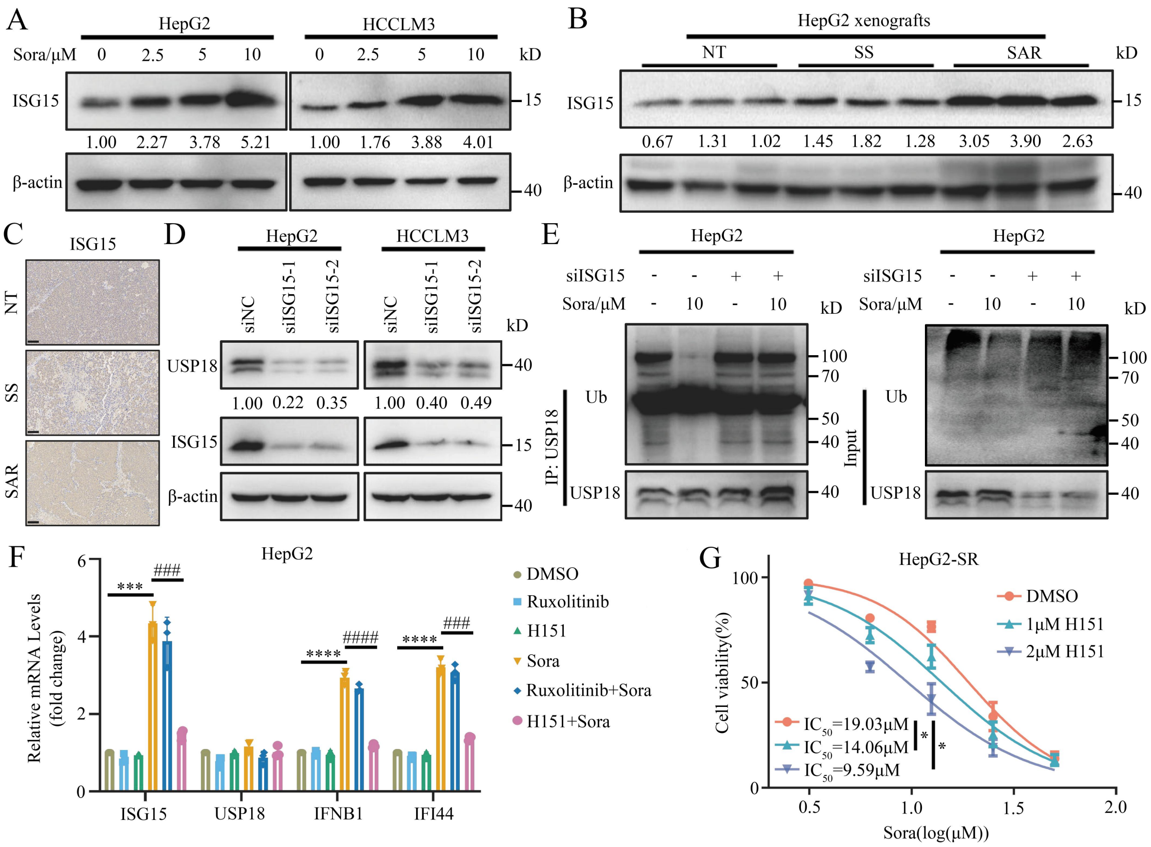

Figure S5. Sorafenib promotes USP18 accumulation via STING/IRF3/ISG15 axis in HCC cells. (A) Protein expression of ISG15 in HCC cells treated with indicated concentrations of sorafenib for 24 h. The band intensities were quantified using Image J and normalized to the control cells treated with DMSO. **(B)** Protein expression of ISG15 in the HepG2 xenografts from the indicated groups. The intensities of bands were analyzed by Image J and normalized to the mean of the corresponding NT group. **(C)** Representative IHC images of ISG15 in excised xenografts from the indicated groups. Scale bars, 50 μm. **(D)** Protein expression of USP18 in HCC-SR cells after transfection with or without ISG15 siRNA for 48 h. The intensities of bands were analyzed by Image J and

normalized to the group transfected with siNC. **(E)** Expression of USP18 ubiquitination in anti-USP18 immunoprecipitation and whole-cell lysates (input) derived from HepG2 cells transfected without or with ISG15 siRNA for 48 h and treated with sorafenib for 24 h. All protein samples were pretreated with 10 μM MG132 for 4 hours prior to collection. *, heavy chain. **(F)** The mRNA expression of downstream target genes regulated by IRF3 in HepG2 cells treated with specified concentrations of Sorafenib, JAK inhibitor (Ruxolitinib), and/or STING inhibitor (H151) for 24 hours was investigated. (***$p < 0.001$ and ****$p < 0.0001$ versus DMSO group, $^{\#\#\#}p < 0.001$ and $^{\#\#\#\#}p < 0.0001$ versus sorafenib (Sora) group, one-way ANOVA test). **(G)** CCK-8 assay. The impact of STING inhibitor (H151) on the susceptibility of HepG2-SR cells to sorafenib (means ± SEM, *$p < 0.05$, one-way ANOVA test).

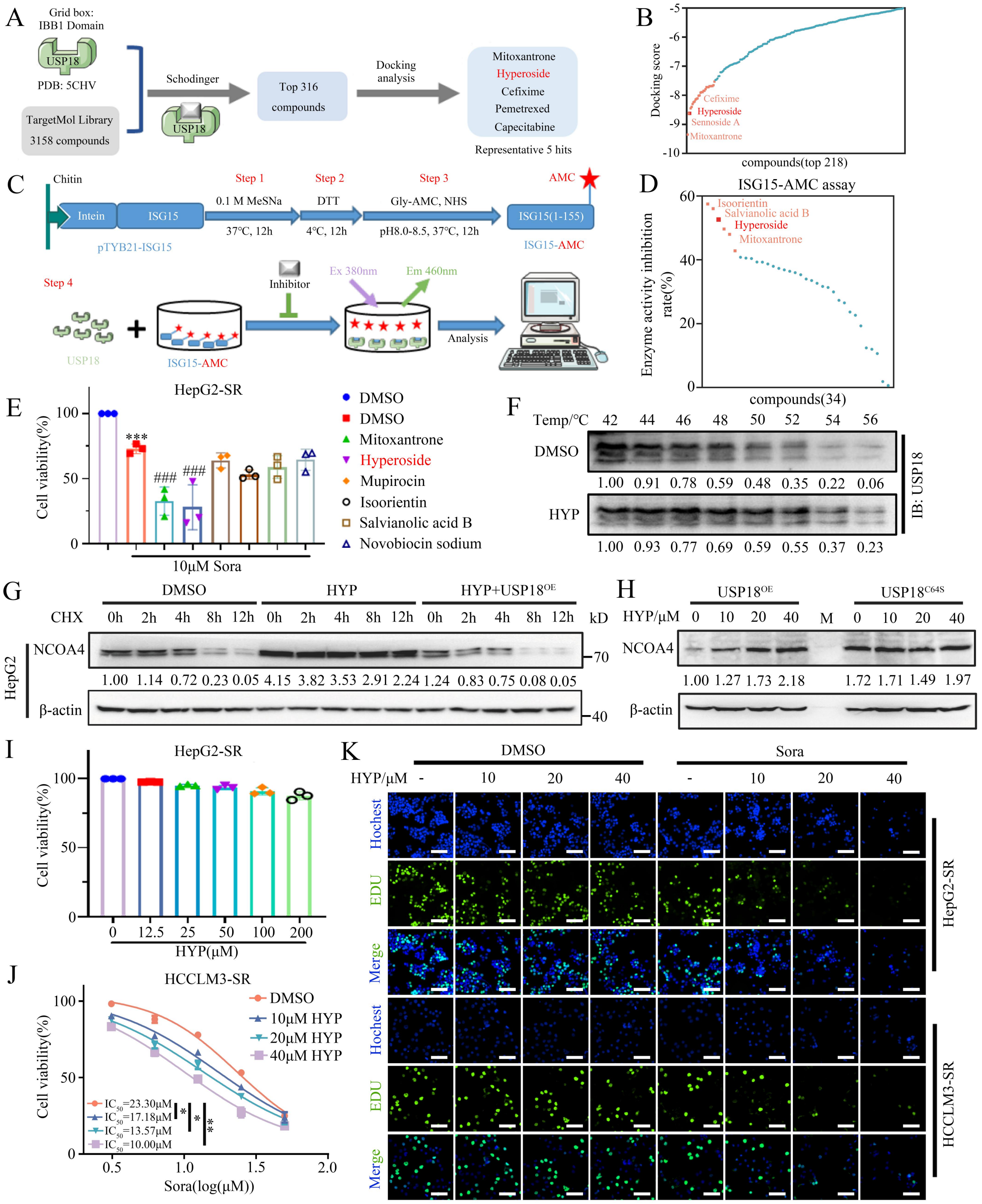

Figure S6. Identification and characterization of HYP as a USP18 inhibitor that directly targets the USP18 IBB1 domain. (A) Schematic overview of the high throughput virtual screening approach used for USP18 inhibitor identification.

(B) The depictions of molecular docking outcomes are illustrated, where compounds demonstrating docking scores below 7.5 are indicated by the rose red squares. **(C)** Schematic diagram of the synthesis and hydrolysis experiments of ISG15-AMC. **(D)** The outcomes of the ISG15-AMC hydrolysis assay. Compounds exhibiting a USP18 enzyme activity inhibition rate exceeding 40% are denoted by rose red dots. **(E)** HepG2-SR cells were treated with 10 μM sorafenib and 20 μM the indicated compounds for 24 h, the cell viability was then measured by CCK-8 assay (means ± SEM, ***p < 0.001 versus DMSO group, $^{###}$p < 0.001 versus sorafenib (Sora) group, one-way ANOVA test). **(F)** Cellular thermal shift assay (CETSA) was used to evaluate the binding between HYP and USP18 in thermodynamic levels. The expression of USP18 was detected by western blot. **(G)** HepG2 cells were transfected with either myc-USP18 plasmid or control plasmid for 48 hours, and then cultured with DMSO or 40 μM HYP for 24 hours before further incubation with CHX for 0, 2, 4, 8, and 12 hours. The NCOA4 protein levels in each group of cells were detected by Western blotting. **(H)** The impact of HYP on the expression level of NCOA4 protein in USP18^{OE} or USP18^{C64S} stable cell lines was investigated using WB analysis. **(I)** CCK-8 assay. The impact of HYP on the cell viability of HepG2-SR cells. **(J)** CCK-8 assay. The impact of HYP on the susceptibility of HCCLM3-SR cells to sorafenib (means ± SEM, *p < 0.05, **p < 0.01, one-way ANOVA test). **(K)** EDU assay. The impact of HYP on the proliferation of HCC-SR cells toward sorafenib treatment. Scale bars, 5 μm.

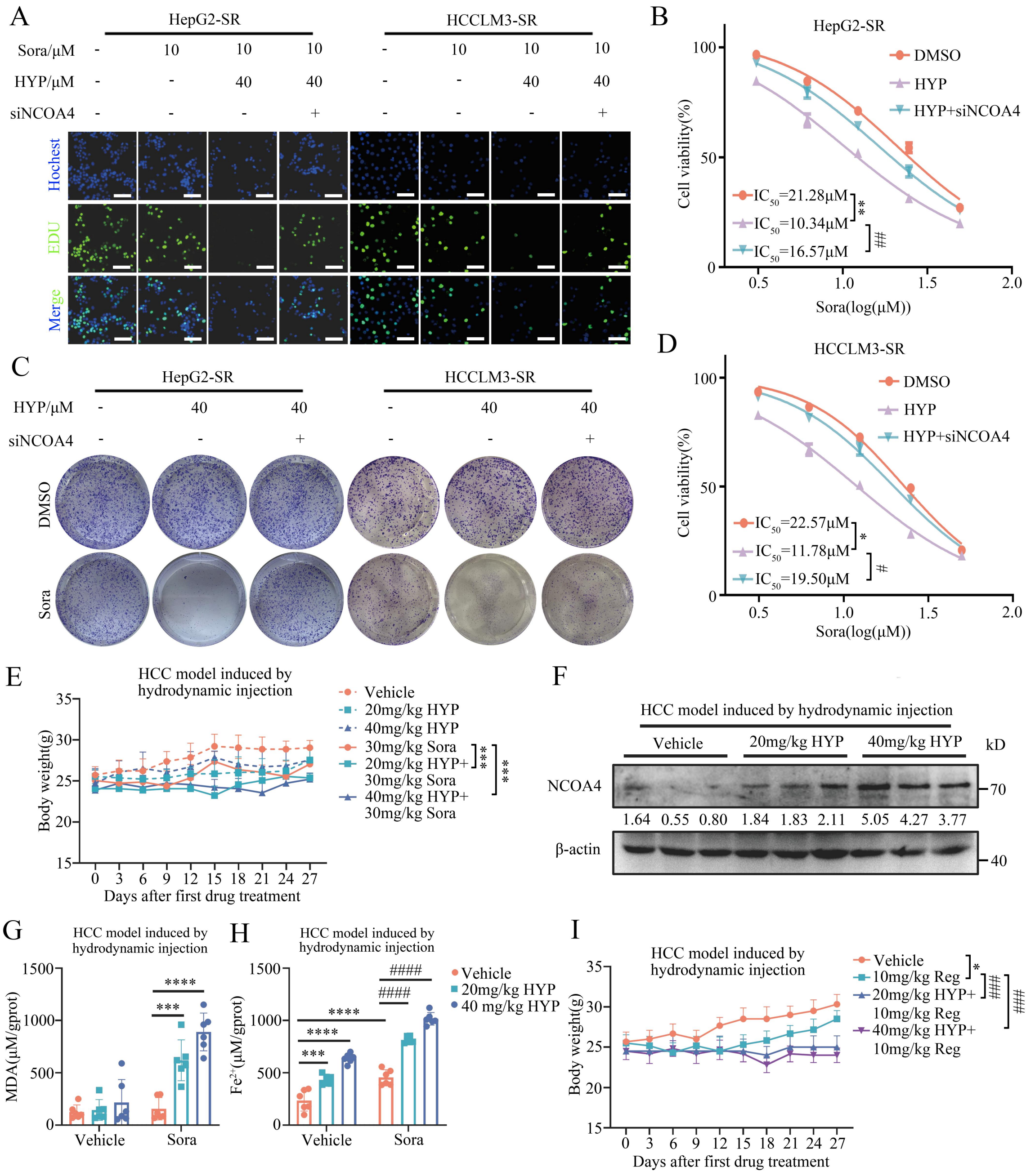

Figure S7. HYP enhances the sensitivity of HCC-SR cells to sorafenib by upregulating NCOA4. (A, B and D) HCC-SR cells were transfected with siNC or siNCOA4 for 48 h and treated with 10 μM sorafenib and/or 40 μM HYP for 24 h. The proliferation cells were analyzed by EDU assay (A). Scale bars, 5 μm. The

cell viability was measured by CCK-8 assay (B and D) (means ± SEM, *p < 0.05, **p < 0.01 versus DMSO, $^{\#}$p < 0.05, $^{\#\#}$p < 0.01 versus HYP, paired student's t-test). **(C)** Colony formation assay. HCC-SR cells were transfected with or without NCOA4 siRNA for 48 h and treated with 10 μM sorafenib and/or 40 μM HYP for 24 h in complete media, washed with PBS, and cultured in complete media for another 14 days. **(E)** The body weights of the indicated groups (means ± SEM, ***p < 0.001 versus 30mg/kg Sora group, one-way ANOVA test). **(F)** Protein expression of NCOA4 in HCC model induced by hydrodynamic injection from the indicated groups. **(G, H)** Detection of MDA content and Fe^{2+} level in the indicated groups of hepatocellular carcinoma model induced by hydrodynamic injection (means ± SEM, ***p < 0.001, ****p < 0.0001 versus Vehicle group, $^{\#\#\#}$p < 0.001, $^{\#\#\#\#}$p < 0.0001 versus 30mg/kg Sora group, one-way ANOVA test). **(I)** The body weights of the indicated groups (means ± SEM, *p < 0.05 versus Vehicle group, $^{\#\#\#}$p < 0.001 versus 30mg/kg Sora group, one-way ANOVA test).
