# Supplementary material for: Targeting USP18 overcomes acquired resistance in hepatocellular carcinoma by regulating NCOA4 deISGylation and ferroptosis
Source: Cell Death Dis. 2025 Jun 13;16(1):448. doi: 10.1038/s41419-025-07772-0 (PMC12166087; doi:10.1038/s41419-025-07772-0)

Figure 1: uncropped Gels and Blots image(s)

C

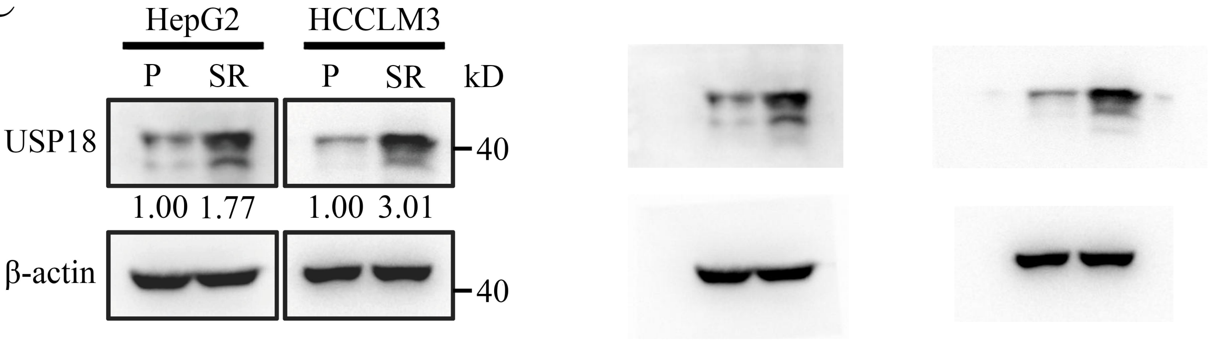

D

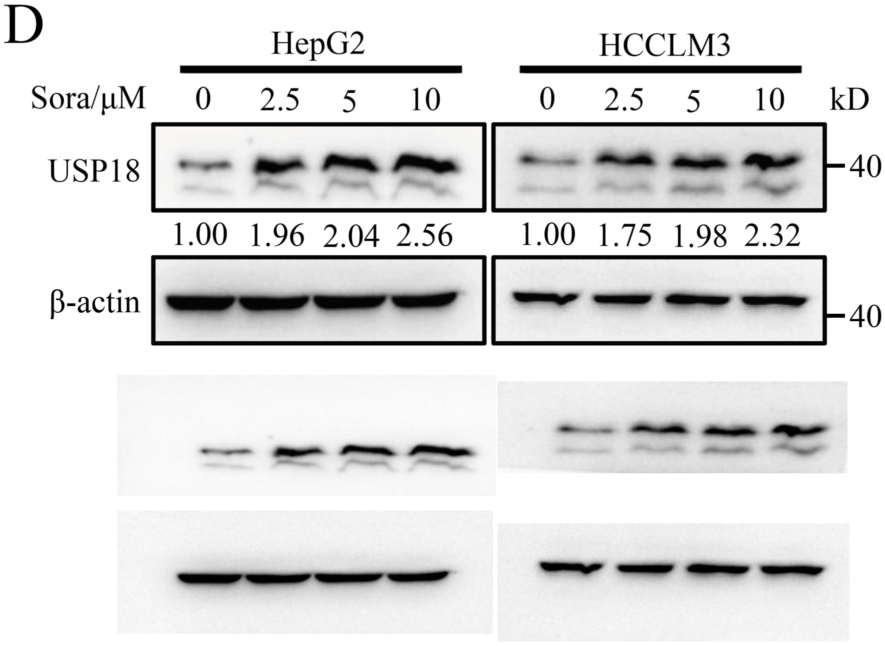

Figure 2: uncropped Gels and Blots image(s)

A

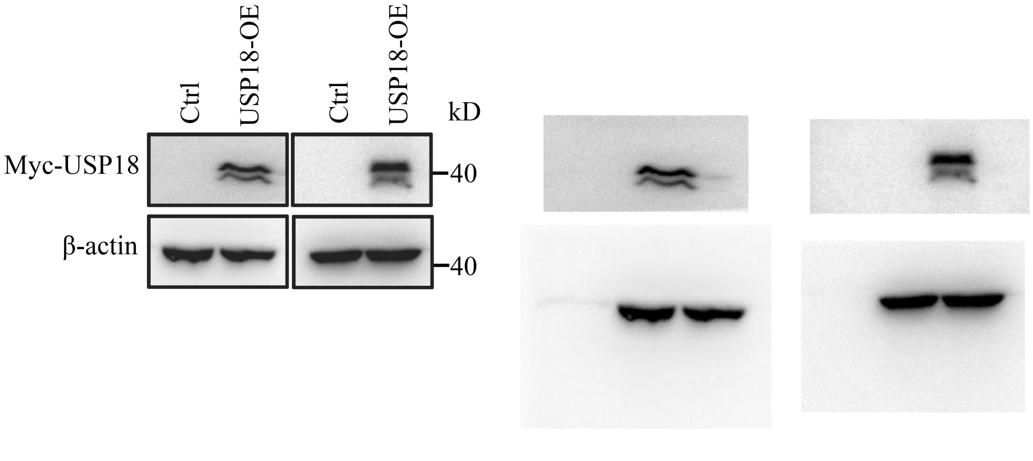

Figure 4: uncropped Gels and Blots image(s)

B

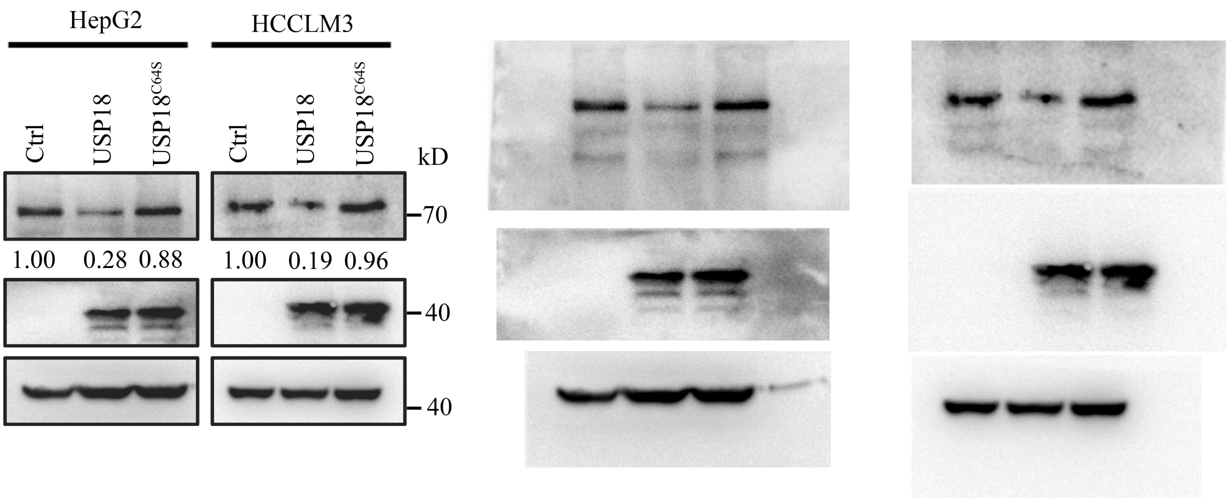

D

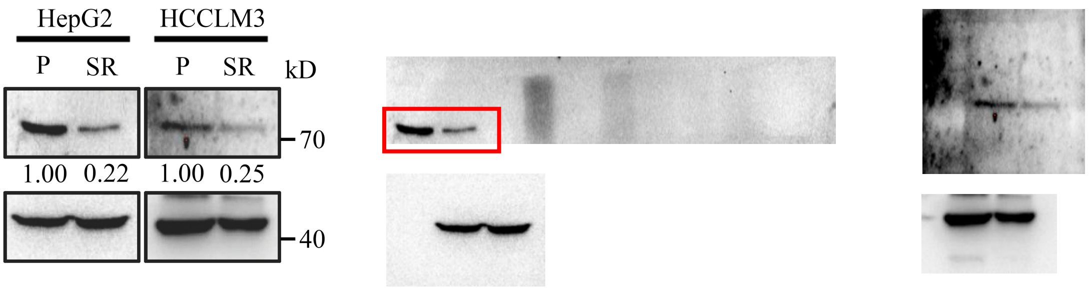

E

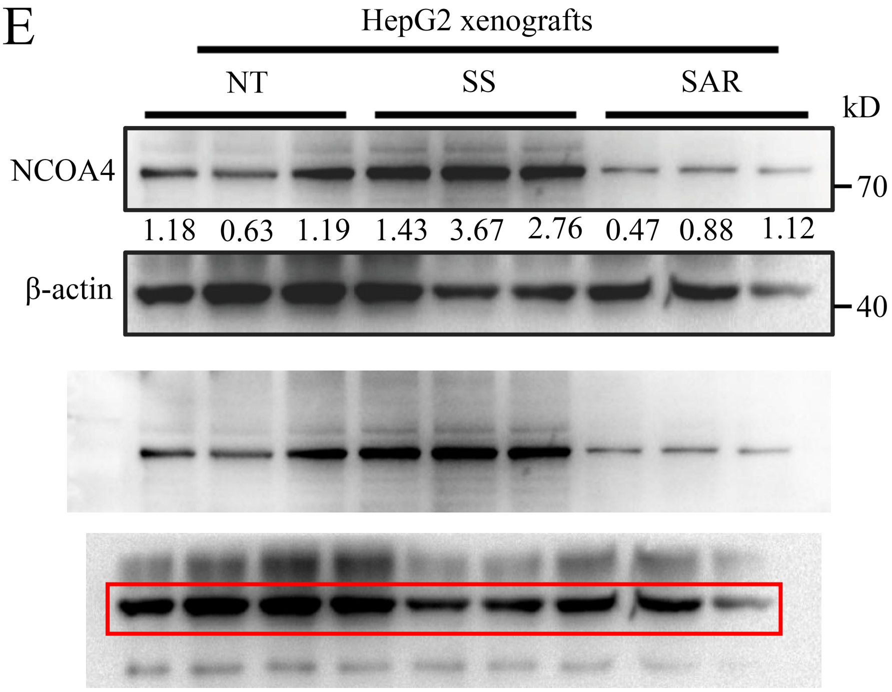

Figure 4: uncropped Gels and Blots image(s)

G

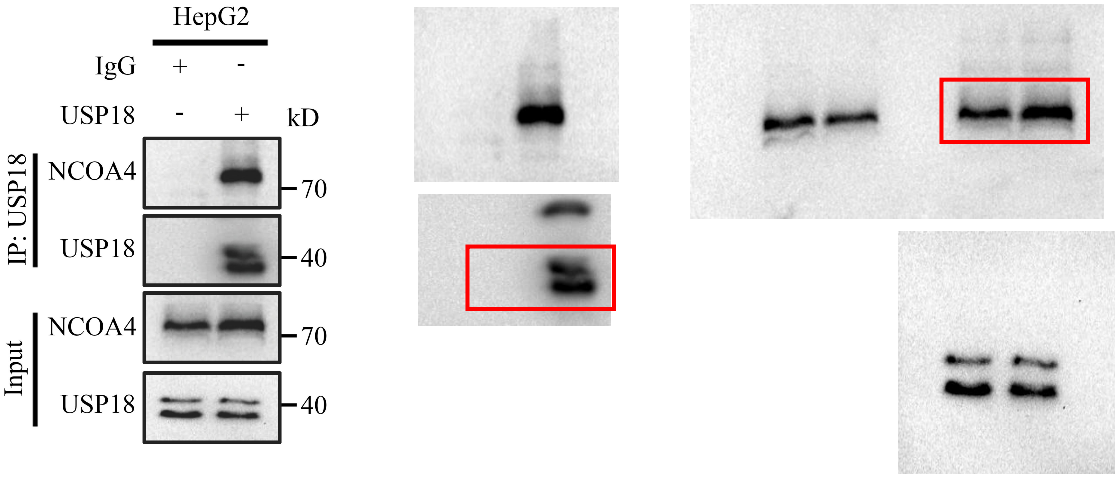

H

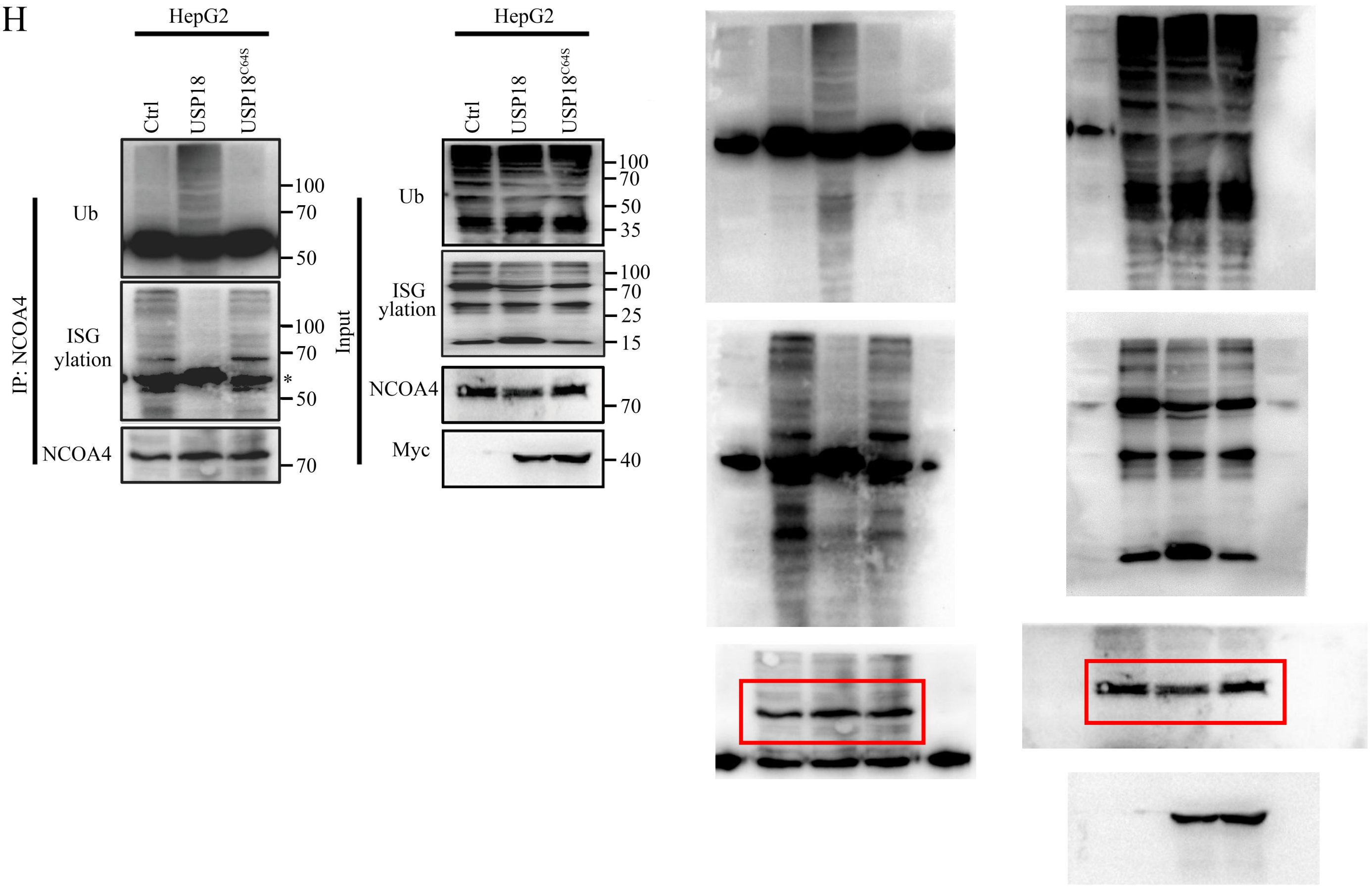

Figure 5: uncropped Gels and Blots image(s)

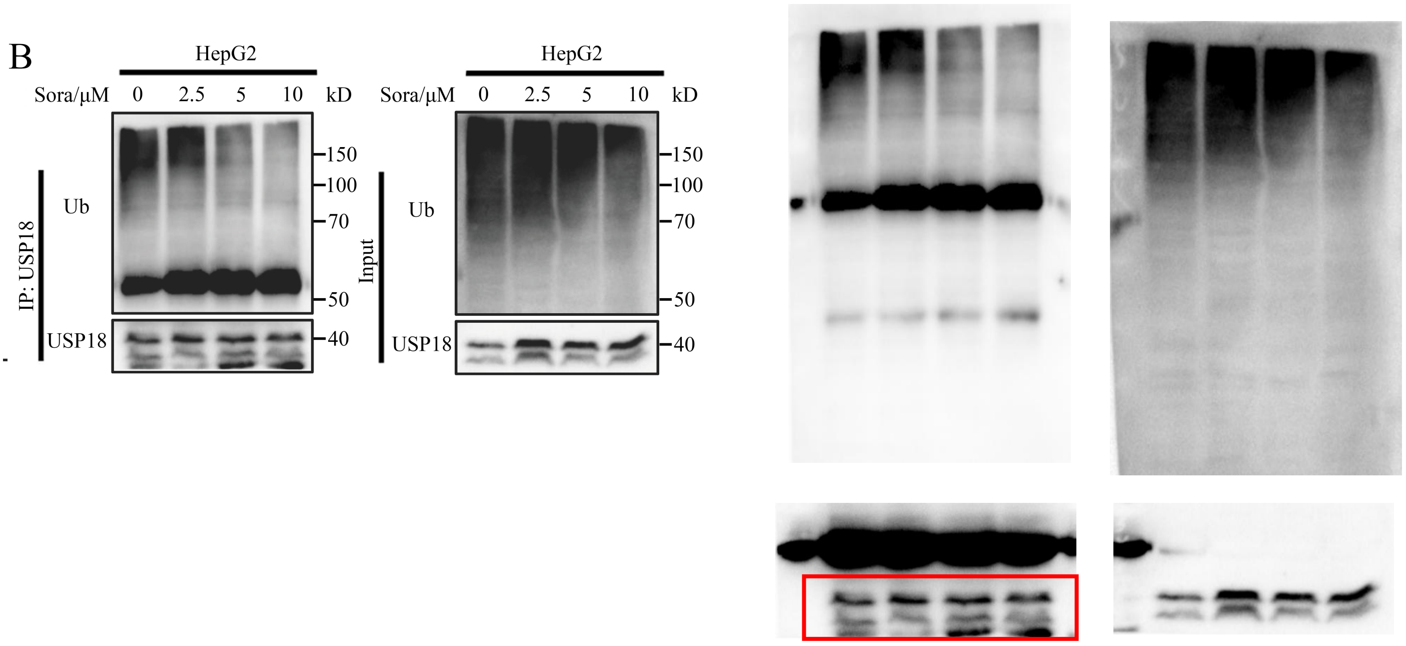

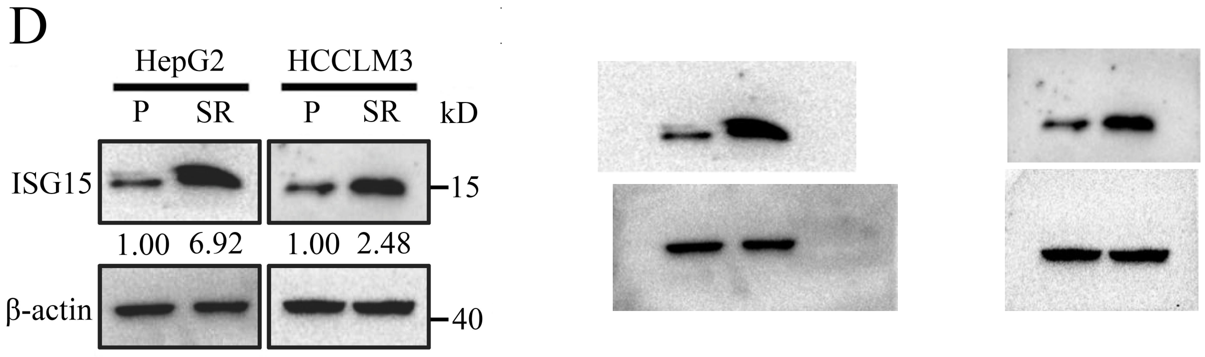

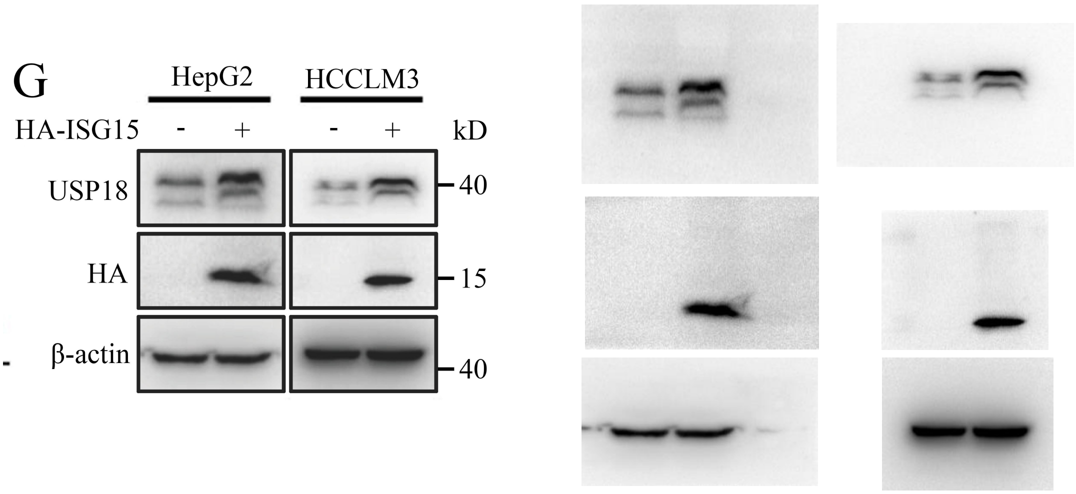

Figure 5: uncropped Gels and Blots image(s)

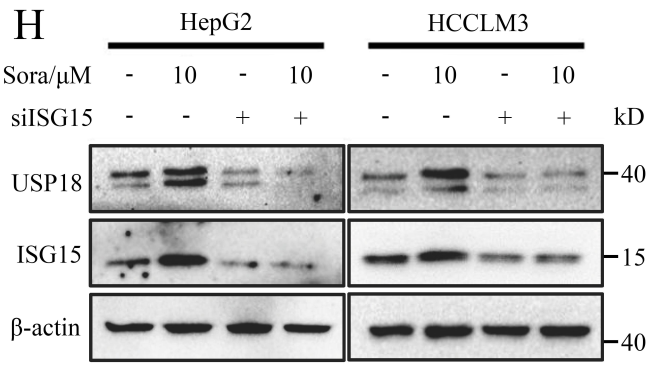

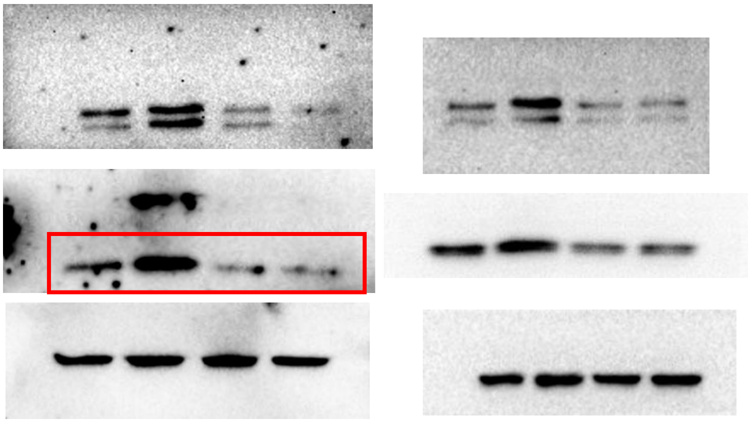

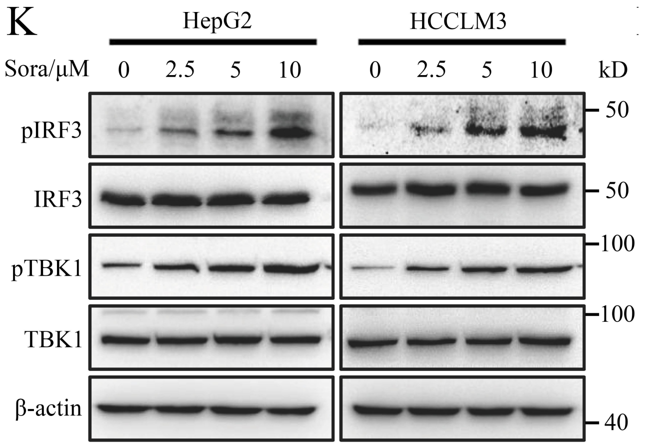

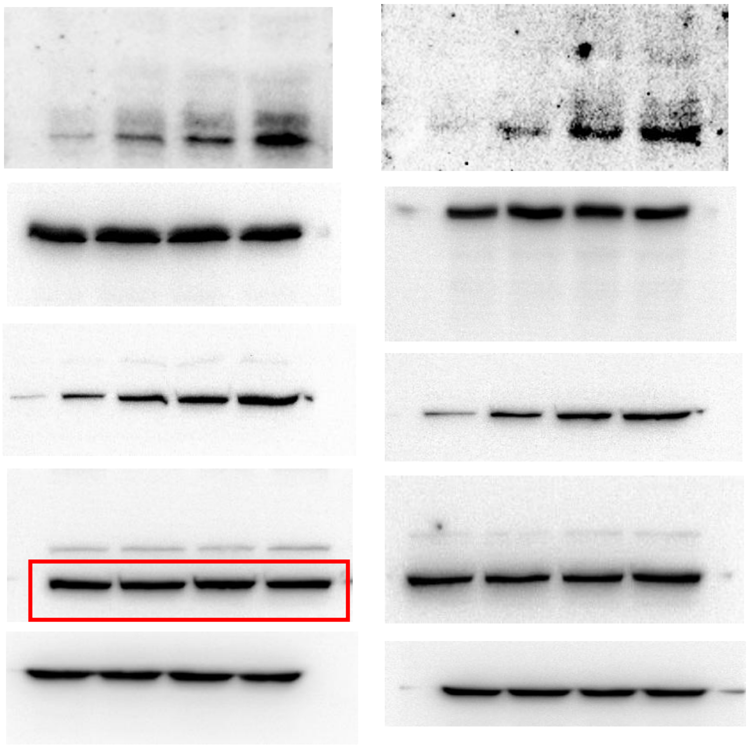

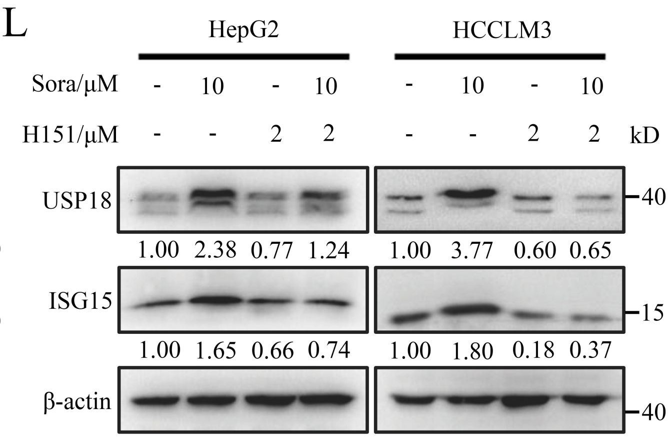

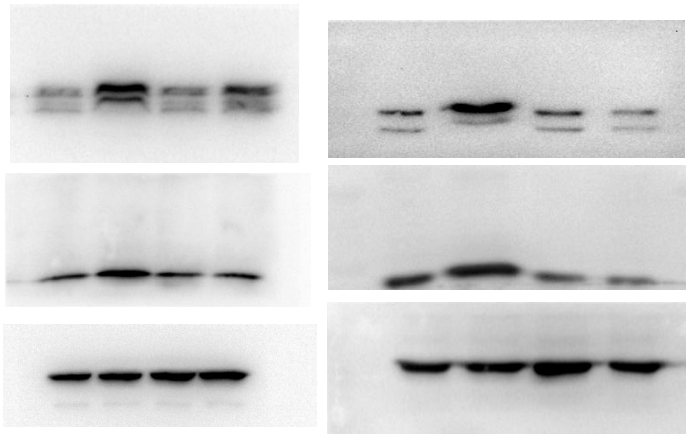

Figure 6: uncropped Gels and Blots image(s)

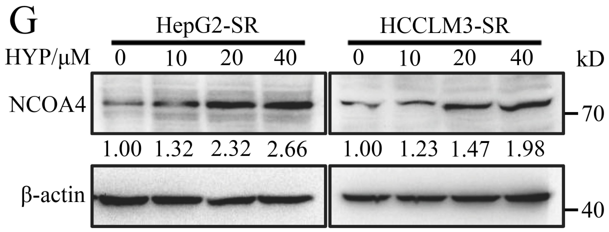

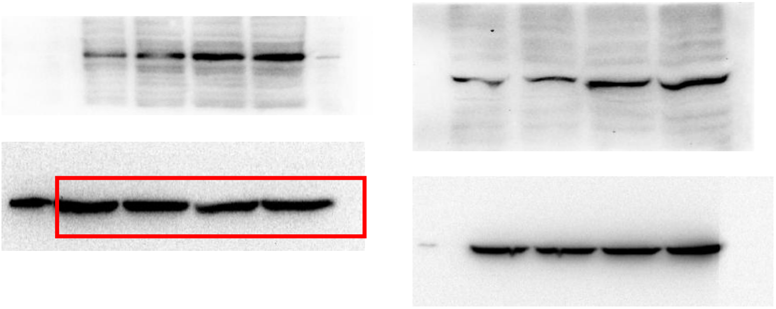

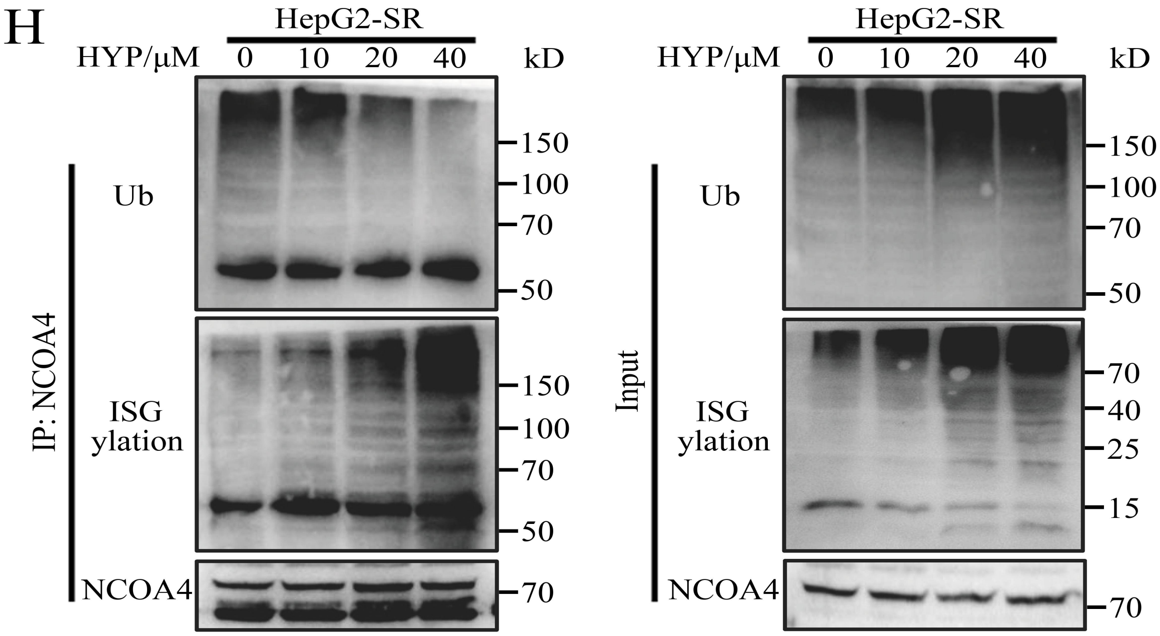

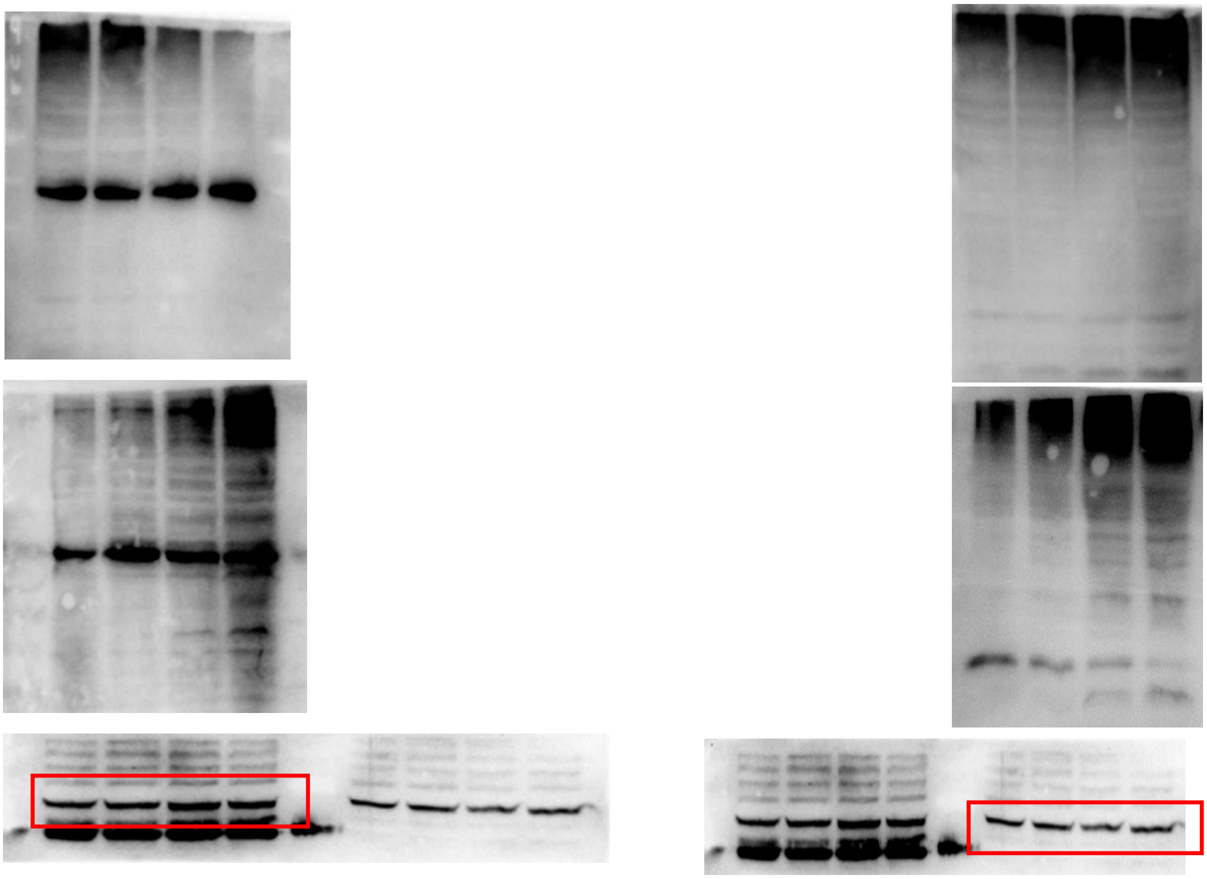

Figure S1: uncropped Gels and Blots image(s)

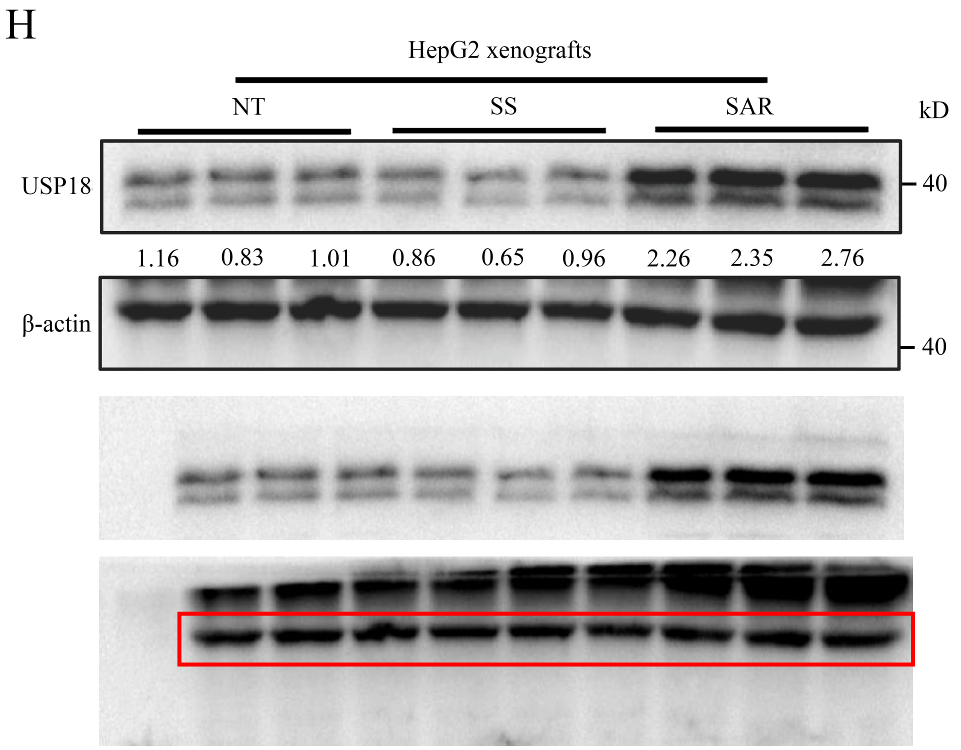

Figure S2: uncropped Gels and Blots image(s)

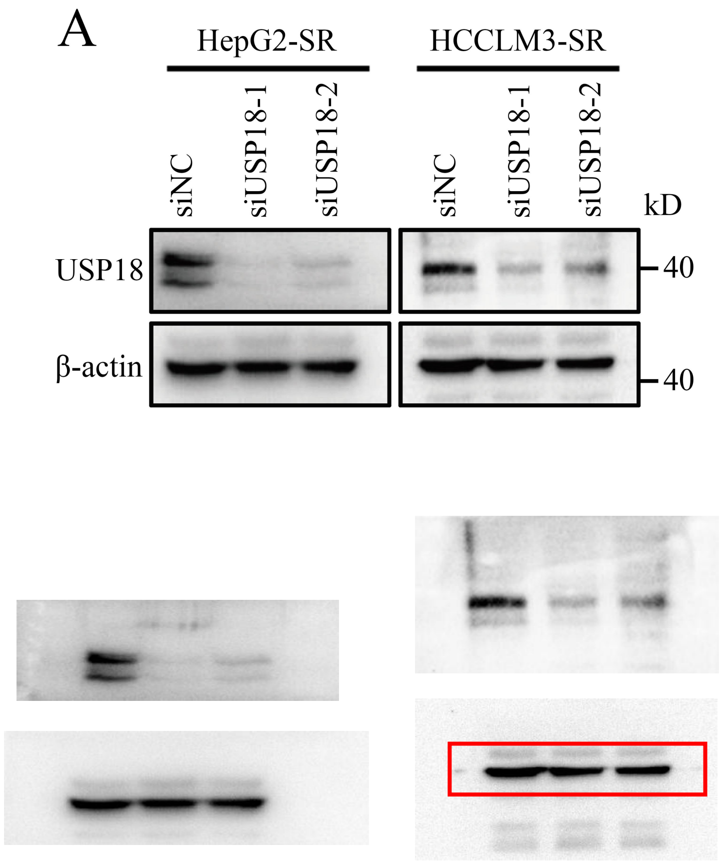

Figure S4: uncropped Gels and Blots image(s)

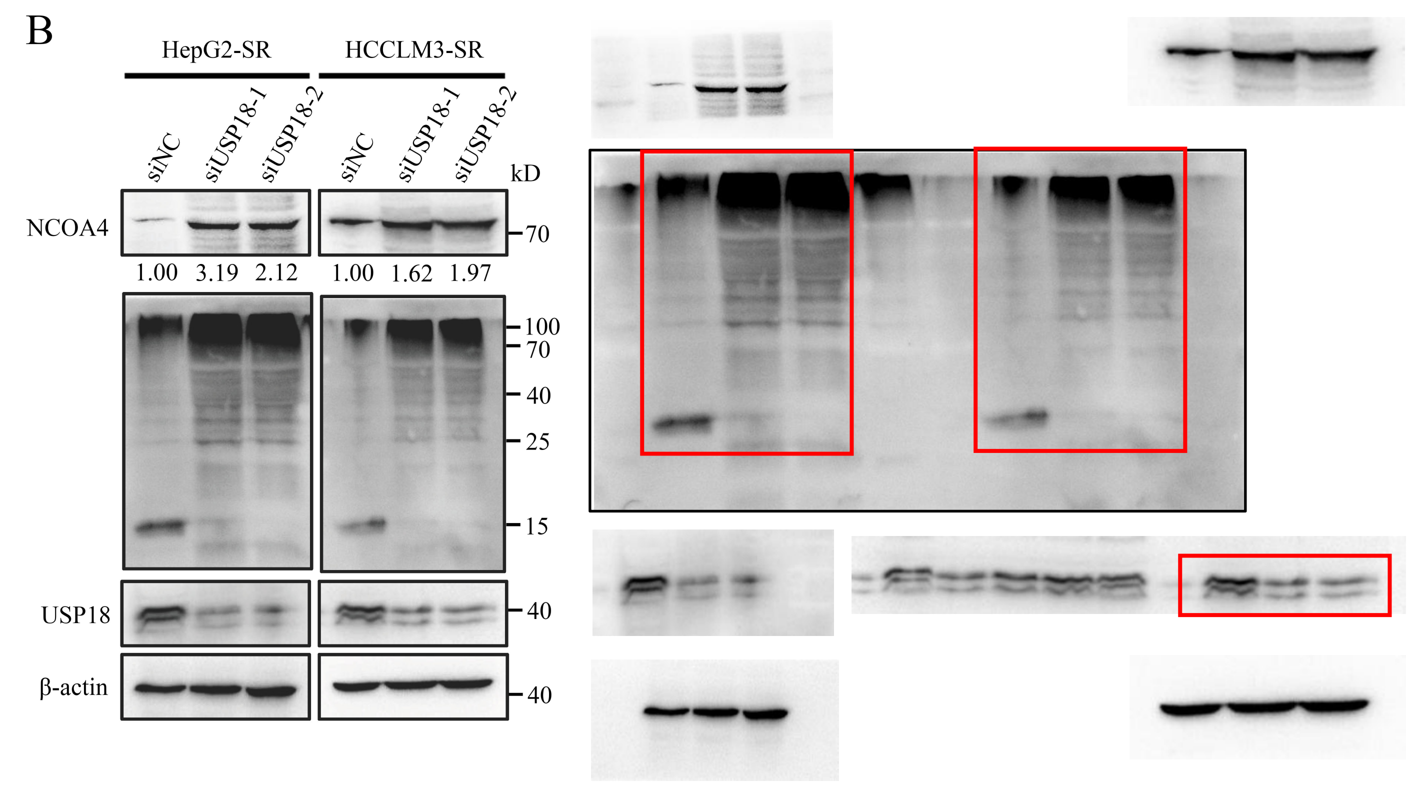

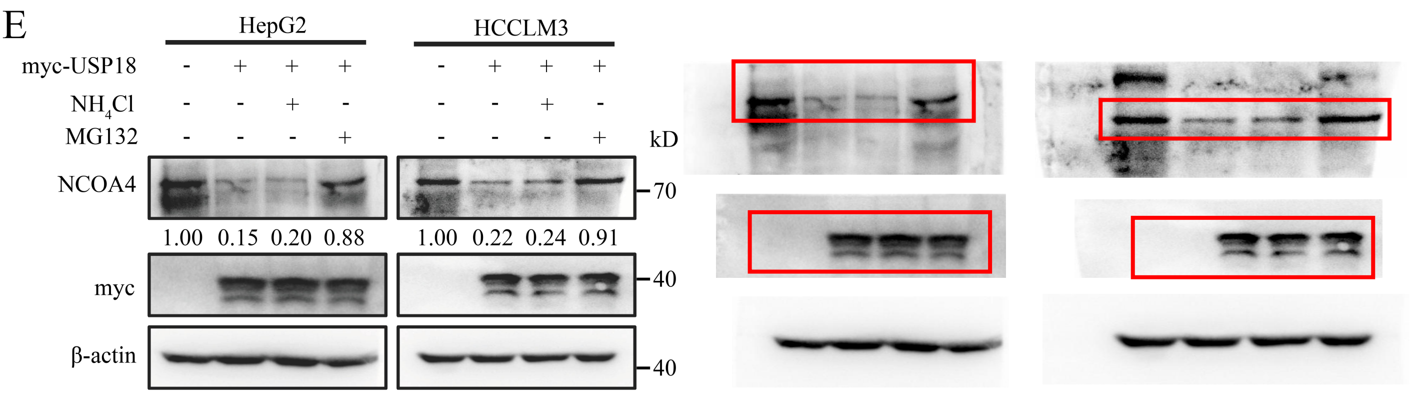

Figure S5: uncropped Gels and Blots image(s)

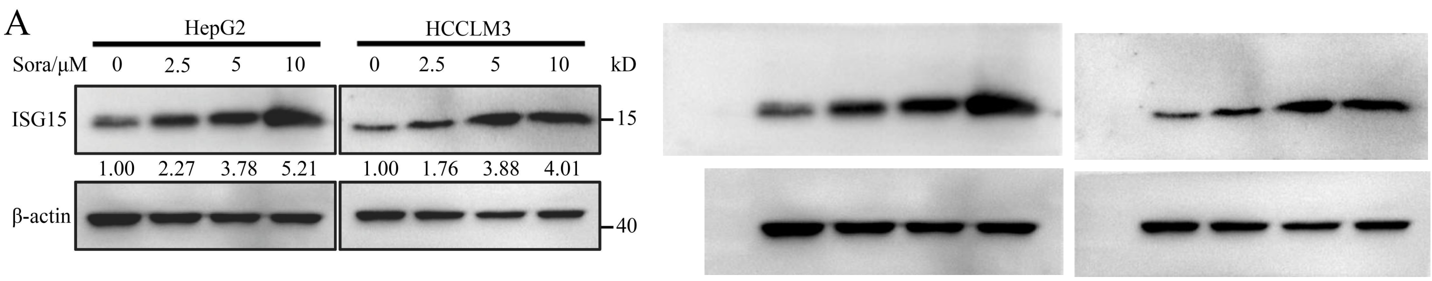

Figure S5: uncropped Gels and Blots image(s)

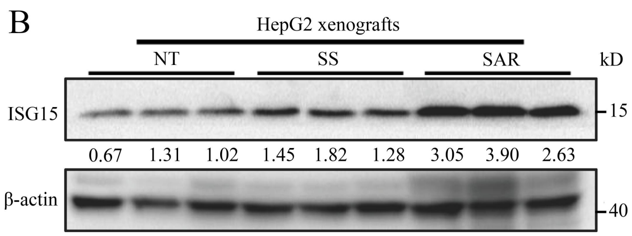

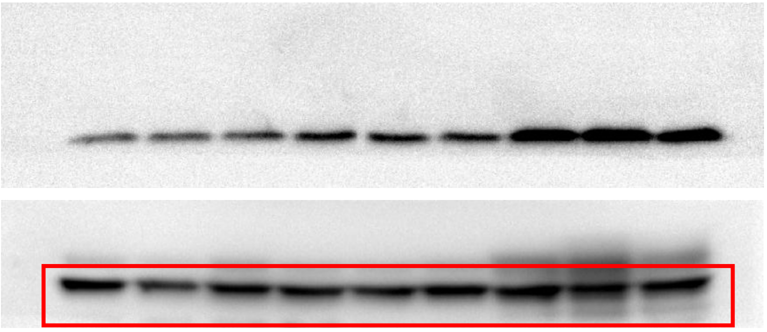

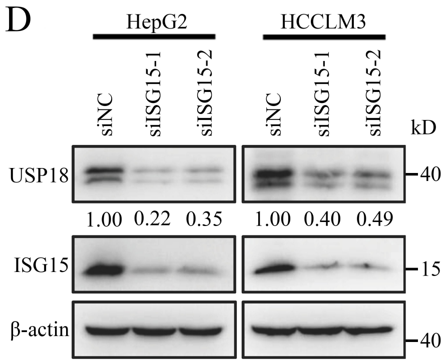

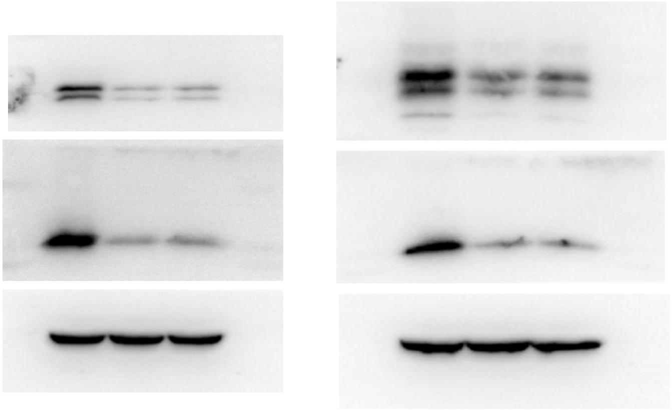

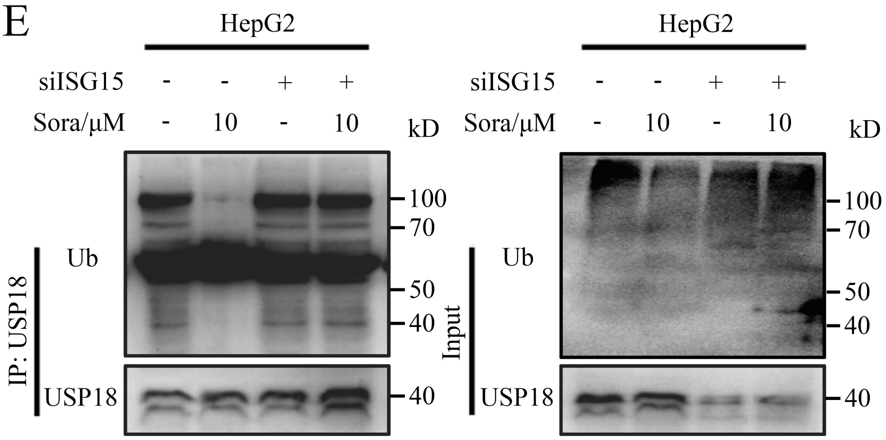

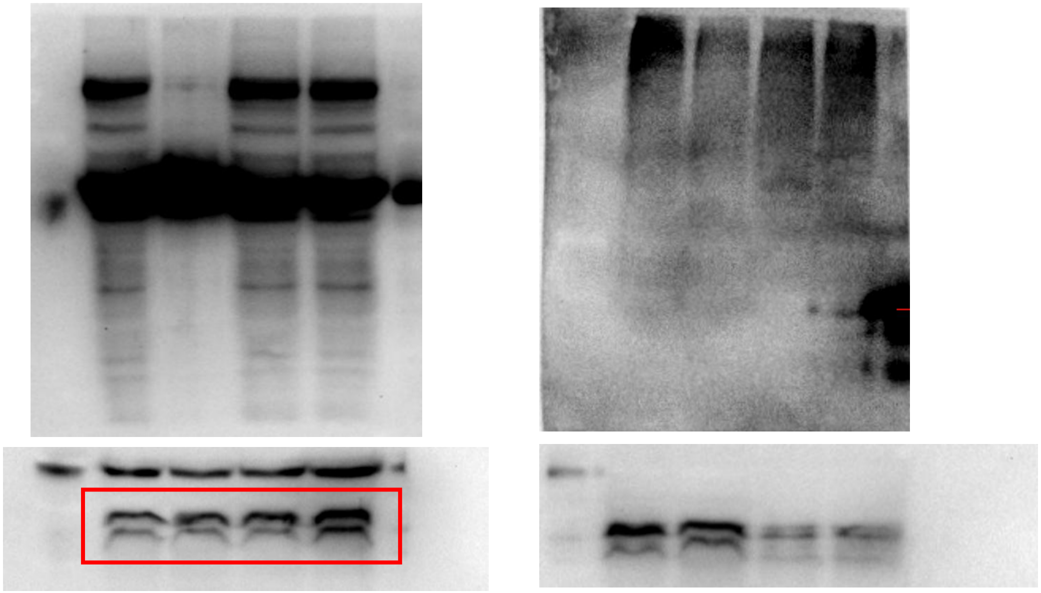

Figure S6: uncropped Gels and Blots image(s)

F

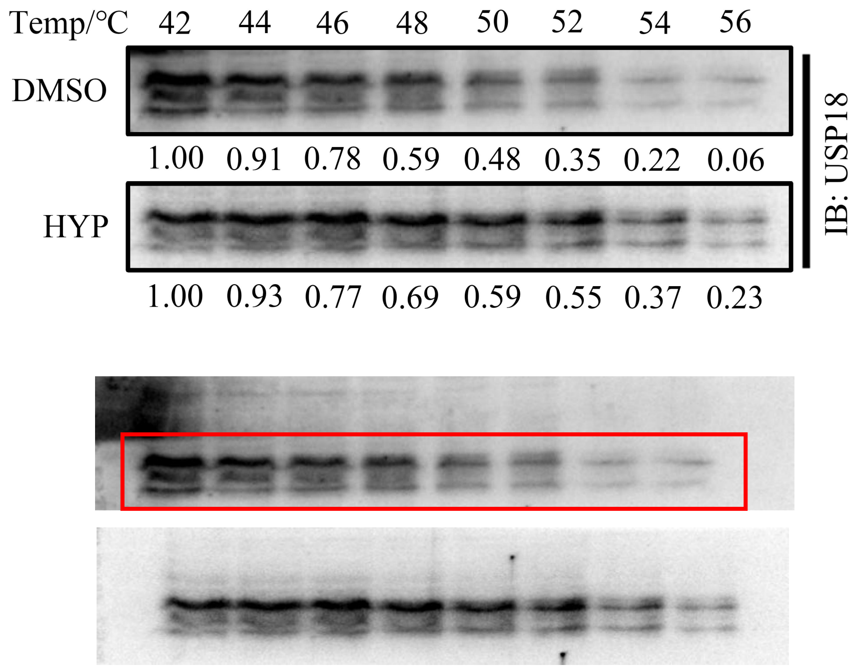

G

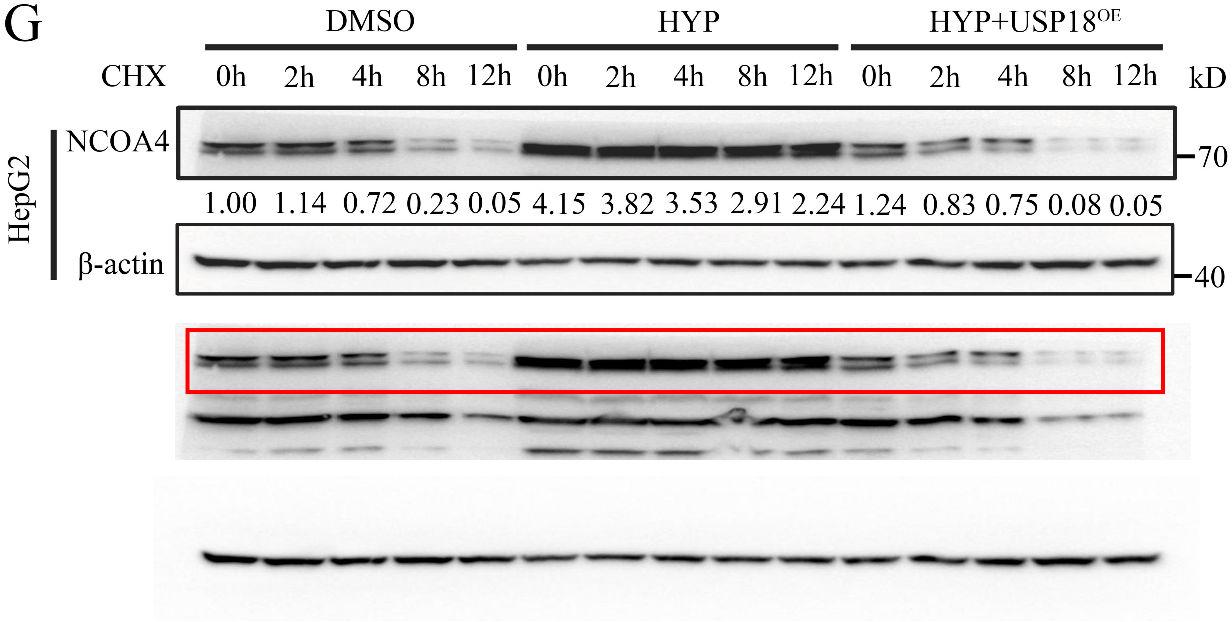

H

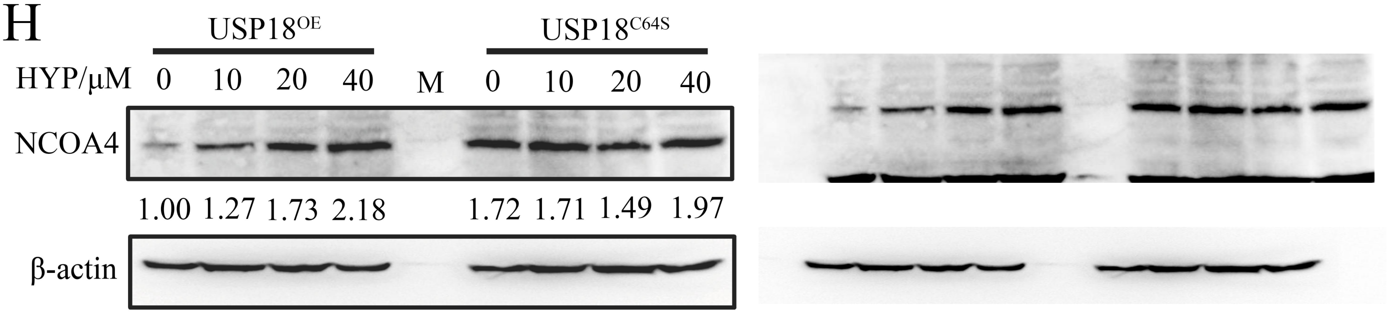

Figure S7: uncropped Gels and Blots image(s)

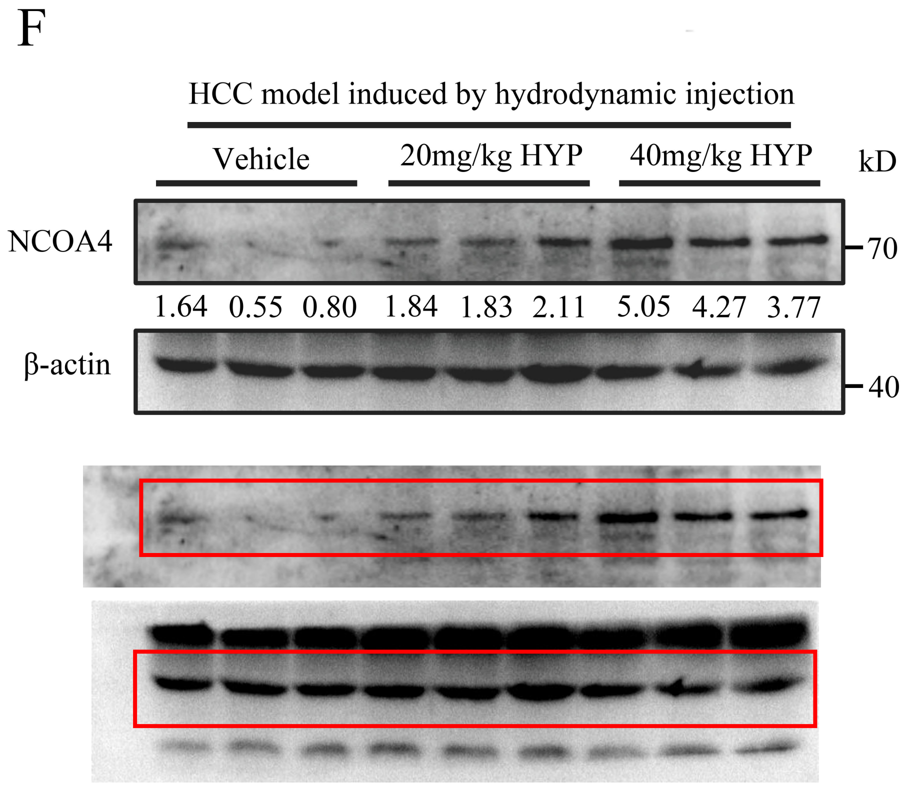

Supplement: Supplementary file 2 — uncropped Gels and Blots image(s)-Cell Death&Disease [file 41419_2025_7772_MOESM2_ESM.pdf]
